# Modelling pyroclastic density currents from a subplinian eruption at La Soufrière de Guadeloupe (West Indies, France)

**DOI:** 10.1007/s00445-020-01411-6

**Published:** 2020-11-13

**Authors:** Tomaso Esposti Ongaro, Jean-Christophe Komorowski, Yoann Legendre, Augusto Neri

**Affiliations:** 1 Istituto Nazionale di Geofisica e Vulcanologia (INGV), Sezione di Pisa, Italy; 2 Université de Paris, Institut de Physique du Globe de Paris (IPGP), CNRS, Paris, France; 3 Bureau de Recherches Géologiques et Minières (BRGM), Petit-Bourg, Guadeloupe France

**Keywords:** La Soufrière de Guadeloupe, Pyroclastic density currents, Subplinian eruption, Numerical simulation, Hazard assessment

## Abstract

We have used a three-dimensional, non-equilibrium multiphase flow numerical model to simulate subplinian eruption scenarios at La Soufrière de Guadeloupe (Lesser Antilles, France). Initial and boundary conditions for computer simulations were set on the basis of independent estimates of eruption source parameters (i.e. mass eruption rate, volatile content, temperature, grain size distribution) from a field reconstruction of the 1530 CE subplinian eruption. This event is here taken as a reference scenario for hazard assessment at La Soufrière de Guadeloupe. A parametric study on eruption source parameters allowed us to quantify their influence on the simulated dynamics and, in particular, the increase of the percentage of column collapse and pyroclastic density current (PDC) intensity, at constant mass eruption rate, with variable vent diameter. Numerical results enabled us to quantify the effects of the proximal morphology on distributing the collapsing mass around the volcano and into deep and long valleys and to estimate the areas invaded by PDCs, their associated temperature and dynamic pressure. Significant impact (temperature > 300 °C and dynamic pressure > 1 kPa) in the inhabited region around the volcano is expected for fully collapsing conditions and mass eruption rates > 2 × 10^7^ kg/s. We thus combine this spatial distribution of temperature and dynamic pressure with an objective consideration of model-related uncertainty to produce preliminary PDC hazard maps for the reference scenario. In such a representation, we identify three areas of varying degree of susceptibility to invasion by PDCs—very likely to be invaded (and highly impacted), susceptible to invasion (and moderately impacted), and unlikely to be invaded (or marginally impacted). The study also raises some key questions about the use of deterministic scenario simulations for hazard assessment, where probability distributions and uncertainties are difficult to estimate. Use of high-performance computing techniques will in part allow us to overcome such difficulties, but the problem remains open in a scientific context where validation of numerical models is still, necessarily, an incomplete and ongoing process. Nevertheless, our findings provide an important contribution to the quantitative assessment of volcanic hazard and risk at La Soufrière de Guadeloupe particularly in the context of the current unrest of the volcano and the need to prepare for a possible future reawakening of the volcano that could culminate in a magmatic explosive eruption.

**Supplementary Information:**

The online version of this article (10.1007/s00445-020-01411-6) contains supplementary material, which is available to authorized users.

## Introduction

Pyroclastic density currents are rapidly moving, gravity-driven flows of gas and hot volcanic particles (ash, lapilli, and blocks) produced during explosive eruptions (Sparks et al. 1978; Druitt 1998; Branney and Kokelaar 2002). They represent extreme hazards at active volcanoes by virtue of their rapid propagation, attaining velocities of up to 60 m/s (e.g. Yamamoto et al. 1993; Loughlin et al. 2002; Komorowski et al. 2015), destructive potential (Spence et al. 2004; Baxter et al. 2005; Jenkins et al. 2010, 2013a), and for the lethal conditions rapidly establishing in the inundated areas (Baxter et al. 2017). Assessing PDC hazards and their associated uncertainty is a challenging task, mostly because of the diversity of PDC generation mechanisms, producing currents with diverse compositions and in a wide range of flow regimes (Fujii and Nakada 1999; Branney and Kokelaar 2002) and by reason of their complex interaction with an incised volcano topography, often leading to unpredictable flow transformations (Fisher 1995; Druitt et al. 2002b; Ogburn et al. 2014; Komorowski et al. 2015). One way to assess hazard and risk at vulnerable sites is through model-based appraisals of PDC invasion and maximum runout, and mapping of hazardous actions in the inundated areas (cf. Calder et al. 2015; Takarada 2017; Lube et al. 2020). Such hazard mapping has been developed both for quiescent volcanoes (e.g. Esposti Ongaro et al. 2008a; Brand et al. 2014; Neri et al. 2015a) and to manage eruptive crises (Wadge and Aspinall 2014; Neri et al. 2015b). Here, we apply a modelling approach to PDC hazard assessment for a subplinian eruption scenario at La Soufrière de Guadeloupe volcano.

**Fig. 1:**
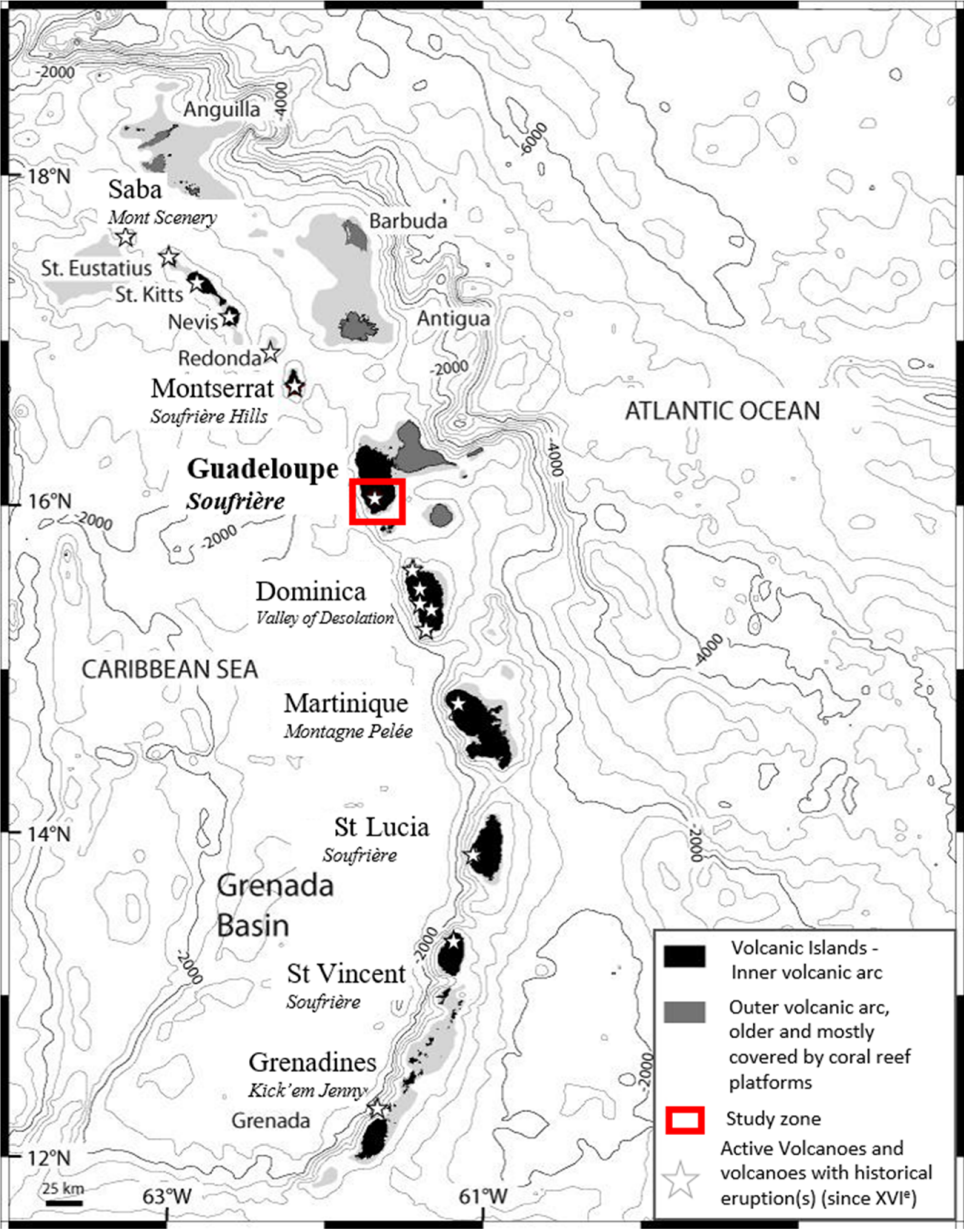
The Lesser Antilles arc. Bathymetry is from Smith and Sandwell (1997). Contour interval is 500 m. Volcanic islands are black, and subaerial coral reef platforms are dark grey. The 100-m depth submarine shelf is light grey (Modified Boudon et al. 2008)

**Fig. 2:**
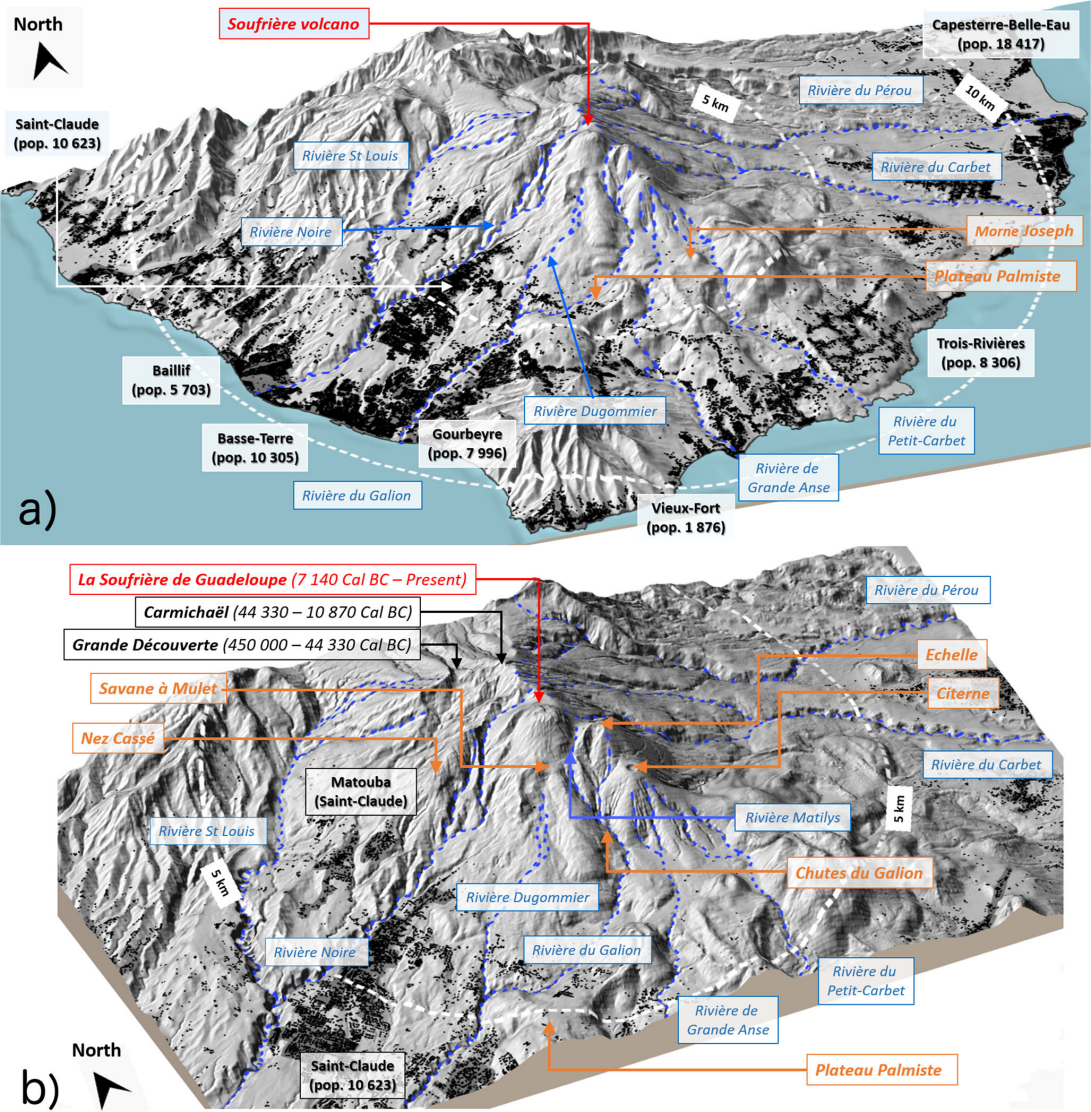
Main topographic elements and locations mentioned in the paper: **a**) Southern Basse-Terre area (study zone; cf. Fig. 1); **b**) zoom on the SDG most proximal area. Demographic data are taken from INSEE (French National Institute of Statistics and Economic Studies) reference data (January 1, 2020). Vertical exaggeration of the topography is by a factor of 1.5. The digital elevation model and building data are from BDAlti® and BDTopo® databases, IGN (Institut Géographique National, France)

The Grande Découverte – Soufrière de Guadeloupe volcanic complex is located on the island of Basse-Terre, in the French Lesser Antilles (Fig. 1). It comprises three stratovolcanoes: Grande Découverte, Carmichael, and La Soufrière de Guadeloupe (SDG) (Fig. 2), which were built during the last 445,000 years (Carlut et al. 2000; Samper et al. 2009). La Soufrière de Guadeloupe is an andesitic composite volcano whose activity over the last 10,000 years has been characterized by a diversity of eruptive styles, including effusive and dome-forming eruptions, explosive phreatic or hydrothermal and magmatic (Vulcanian to plinian) eruptions, and numerous flank collapse events (Komorowski et al. 2002, 2005; Boudon et al. 2007; Legendre 2012). The most recent magmatic subplinian eruption dates from 1530 CE (Boudon et al. 2008; Komorowski et al. 2008), and a smaller magmatic (Vulcanian to subplinian) eruption took place in 1657 CE (Legendre 2012; Rosas-Carbajal et al. 2016). The historical activity since the 1657 eruption has been characterized by minor (1690, 1812, and 1956) and major (1797–1798, 1836–1837, and 1976–1977) non-magmatic (phreatic) eruptions. These eruptions have taken place from fractures and vents on SDG’s lava dome (Feuillard et al. 1983; Boudon et al. 1988; Komorowski et al. 2005; Rosas-Carbajal et al. 2016). The last and most violent phreatic eruption occurred in 1976–1977 and forced the evacuation of about 73,600 people for up to 4 months. Although it did not evolve into a magmatic eruption, geophysical and geochemical evidence supported its interpretation as a shallow intrusion that did not feed an eruption (Feuillard et al. 1983; Villemant et al. 2005). This failed magmatic eruption (Moran et al. 2011) involved a small-volume magma intrusion that ascended from the 6–8.5-km-deep magma reservoir (Pichavant et al. 2018) and stagnated at shallower depth, pressurizing the hydrothermal system at a depth of about 500 m below the summit (Feuillard et al. 1983; Boudon et al. 1988; Villemant et al. 2005; Hincks et al. 2014).

Although interpretation of the eruptive history of SDG has been particularly difficult on account of erosion and alteration processes that are particularly intense under the tropical climate, geological studies suggest there have been several magmatic explosive eruptions in the last 10,000 years including at least two subplinian VEI 2–3 and six Plinian VEI 4 (Komorowski et al. 2005; Legendre 2012). The 1530 CE eruption is representative of a typical subplinian (VEI 3) magmatic explosive eruption at SDG and is interpreted to be the most credible eruptive scenario for a future event (Boudon et al. 2008; Komorowski et al. 2008).

**Fig. 3:**
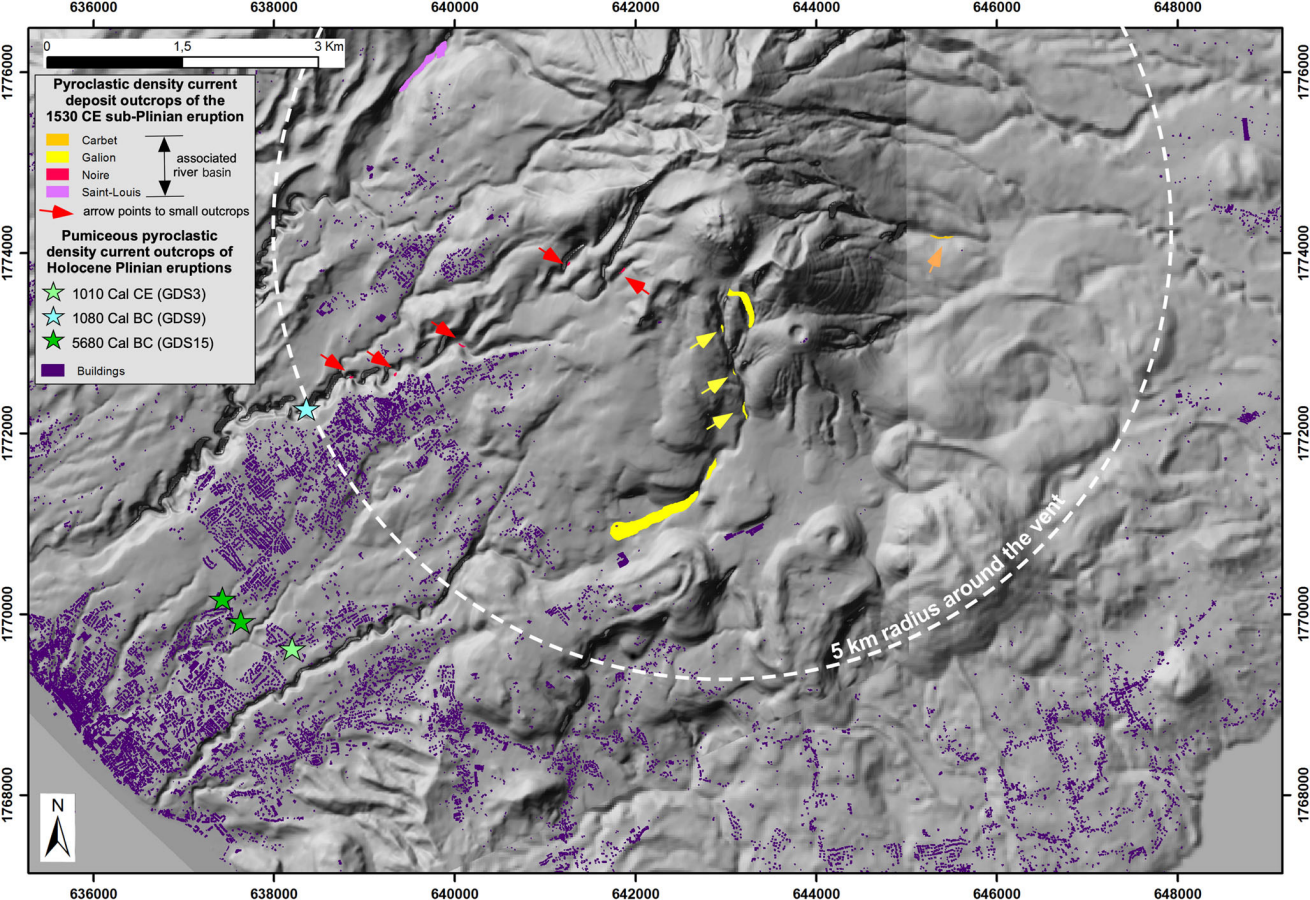
Map of PDC outcrops and inferred eroded PDC deposits from the last major magmatic subplinian eruption at La Soufrière de Guadeloupe in 1530 CE (modified after Boudon et al. 2008; Komorowski et al. 2008; Legendre 2012; Komorowski et al. 2013). The digital elevation model and building data are from BDAlti® and BDTopo® databases, IGN

The 1530 CE eruption began with phreatic explosions followed by partial collapse of the edifice that emplaced a debris avalanche (Komorowski et al. 2002, 2005; Boudon et al. 2008), which travelled at least 9 km in the South-West direction and reached the sea at *Basse-Terre* (all places referred to herein are located on Fig. 2). It then evolved into a short (ca. 1 h long) subplinian phase (with an intensity of between 5 × 10^6^ and 2 × 10^7^ kg/s; Komorowski et al. 2008, 2013). This phase produced coarse pumice and scoria fallout from a column inferred to have reached 16 to 18 km in height, as well as pumice and scoria-rich PDCs from column collapse. Deposits from PDCs are found in a very few poorly preserved and ephemeral exposures scattered in different valleys around SDG’s dome, notably in the *Rivière du Carbet* (East), *Rivière Noire* (West), *Rivière Saint-Louis* (North-West), and in the *Rivière du Galion* (South-Southwest), up to maximum distances of about 6–7 km (Boudon et al. 2008; Fig. 3). Following the subplinian phase, a short-lived period of violent strombolian activity occurred producing stratified scoria fall layers mostly to the North-East of the vent. The final phase of the eruption produced an andesite lava dome (ca. 50 × 10^6^ m^3^) within the depression left by the partial edifice collapse at the onset of the eruption (Komorowski et al. 2002, 2005; Boudon et al. 2008). Interpretation of field data, considering erosion and weathering processes, the short distance between SDG’s summit and the coastline (9 km), and the unknown mass of fine-grained fallout deposits in the sea and thus unaccounted for, suggests that PDC deposits from the 1530 CE eruption account for 57% of the total mass erupted (Komorowski et al. 2013). Outcrops of pumice PDCs from older plinian (VEI 4) eruptions of SDG have been identified at distances of about 8 km from the vent (green stars in Fig. 3). It is likely that they might have reached further given the intense erosion that has affected the deposits since their emplacement (Legendre 2012; Komorowski et al. 2013).

Owing to the complexity of the eruptive sequences of SDG and difficulties in reconstructing a complete eruptive record due to dense vegetation, erosion, and alteration processes (Legendre 2012), the quantitative assessment of volcanic hazards in Guadeloupe is still an ongoing, challenging, and urgent task. Indeed, seismic, fumarolic, and thermal unrest at SDG has been slowly increasing since 1992 (Komorowski et al. 2005; OVSG-IPGP 1999-2020). In April 2018, the unrest reached its highest level since the end of the 1976–1977 failed magmatic eruption (Moretti et al. 2020; OVSG-IPGP 1999–2020). Although the alert level has remained at yellow (vigilance), the increasing unrest has prompted reinforced surveillance by the Volcanological and Seismological Observatory of Guadeloupe and the decision by authorities to implement an exclusion zone for the general public to the most active areas of the summit (Préfet de la Région Guadeloupe 2019).

This work thus contributes to a volcanic risk assessment strategy in Guadeloupe initiated several years ago (Komorowski et al. 2005, 2008, 2013; Hincks et al. 2014; Legendre 2012; Peruzzetto et al. 2019; Leone et al. 2019), with the first integrated hazard map for SDG being produced by Komorowski et al. (2005). Subsequently a first attempt to characterize the eruptive behaviour in a systematic way was carried out by Komorowski et al. (2008), who defined a logical event tree for magmatic unrest and eruptions. Using this framework, and by means of a combined field study and numerical simulations, Komorowski et al. (2008) analysed the hazards associated to the tephra fallout phase in a subplinian scenario similar to 1530 CE event. Considering the recurrence of PDCs in the volcanic history of SDG, and the evidence of PDC deposits in urbanized areas (Legendre 2012), it seems very likely that for such a future magmatic eruption, PDCs could affect, directly or indirectly, a very large part of the South of the island of Basse-Terre, where some 70,000 people live. Given the topography of the area and the geometry of rivers that drain the volcano and reach the inhabited areas, one of the most important issues of the hazard and impact assessment is to model the influence of the topography on the mobility and dynamics of the PDCs and the associated inundation areas.

We thus approach the dynamics of PDCs, and the associated hazards, by numerically simulating in 3D and time an eruptive scenario characterized by the collapse of a volcanic column and subsequent PDC propagation over the incised topography of the volcano. In particular, we aim at understanding which eruptive conditions would be able to generate PDCs attaining the distances where outcrops from the 1530 CE eruption have been found (Fig. 3). In addition, because poor deposit preservation makes it impossible to obtain a homogeneous picture of the areas potentially inundated by PDCs, we use numerical modelling to understand how the volcano summit topography distributes the mass of material from a collapsing column around the vent and into valleys. Finally, quantifying how PDCs would impact the inhabited zone on the volcano flanks has important implications in terms of hazard assessment, risk mitigation, and crisis response in the event of a future eruption.

## Subplinian eruption modelling and hazard assessment

Following Komorowski et al. (2008) and Boudon et al. (2008), the last magmatic eruption of SDG in 1530 CE is taken as a reference scenario for assessing hazards associated with PDC emplacement. The eruption followed the partial collapse of the edifice that resulted in the formation of a 500-m-wide horseshoe-shaped collapse structure open to the South and South-West and the emplacement of a debris avalanche deposit (Boudon et al. 2008; Komorowski et al. 2008; Legendre 2012). However, we here only consider the subplinian phase and, in particular, the stage of collapse of the volcanic column and generation of PDCs.

Defining the source parameters for an eruptive scenario based only on the interpretation of partial sedimentological records is a challenging task. Plinian and subplinian eruptions are characterised by a phase of formation of the convective plume and umbrella cloud, with associated tephra fallout deposits, and by simultaneous (or alternating) phases of column collapse and PDCs (Cioni et al. 2002). The height reached in the atmosphere by the convective column and umbrella can be estimated from the inversion of observable field data (e.g. maximum clast size isopleths of the fallout deposit or remote sensing data) using simplified models (e.g. Pyle 1989; Scott et al. 1996; Mastin et al. 2009; Burden et al. 2011; Bonadonna and Costa 2012; Biass et al. 2019) or numerical methods (Cerminara et al. 2015). For the 1530 CE eruption, the column height has been estimated at between 9 and 12 km from tephra fall deposits by Komorowski et al. (2008). This corresponds to an estimated peak mass eruption rate of between 5.5 × 10^6^ and 1.3 × 10^7^ kg/s, i.e. in the range of subplinian eruptions (Newhall and Self 1982; cf. Cioni et al. 2002). With new field data (Legendre 2012), the column height has been determined to have reached 16 to 18 km, for a mass eruption rate on the order of 7 × 10^6^–2 × 10^7^ kg/s, a volumetric flux of 4–7 × 10^3^ m^3^/s, and an estimated minimal eruption duration of 0.7 h (Komorowski et al. 2013). For realistic eruption conditions (volatile content between 2 and 5 wt.% and temperatures between 950 and 1100 °C), both one-dimensional (Wilson et al. 1980; Woods 1988; Ishimine 2006; Carazzo et al. 2010) and three-dimensional numerical models (Suzuki and Koyaguchi 2012; Koyaguchi and Suzuki 2018) show that mass eruption rates in this range lie at the threshold between a convective and collapsing plume regime, which can be termed a *transitional* or *oscillating* regime (Neri and Dobran 1994; Di Muro et al. 2004; Suzuki and Koyaguchi 2012). To reconstruct the mass eruption rate at the time of collapse during transitional regimes, we have assumed, based on Wilson et al. (1980), that this is equal to the maximum intensity achieved during the convective phase. However, the numerical investigations of Trolese et al. (2019) demonstrate that plume height is strongly reduced during partial collapse episodes, so that the mass eruption rate might be underestimated. Moreover, in some situations full collapse (*boiling over* or *fountain collapse*; Fisher and Heiken 1982; Druitt et al. 2002a; Sulpizio et al. 2014) of a subplinian column can be triggered by the downward collapse of the edifice into an emptying chamber to form a summit caldera. This might imply a significant increase of the eruption intensity (cf. Marti et al. 2000; Cioni et al. 2002) due to the sudden enlargement of the vent (cf. Wilson et al. 1980). Although there is no clear evidence for a summit caldera collapse at SDG during the 1530 CE eruption, a sudden enlargement of the vent might have resulted as a consequence of an initial phase of partial lateral flank collapse. Moreover, geophysical imaging (i.e. electric conductivity and spontaneous potential; Brothelande et al. 2014; Rosas-Carbajal et al. 2016) indicate the presence of an arcuate vertical structure to the South-West and South of the current dome that may mark the relict margins of the explosion crater associated with the eruption within which the dome grew at the end of the eruption (Boudon et al. 2008). Overall, the structural features surrounding the current dome show a combination of an explosion crater and edifice collapse structure that is roughly circular and about 900 m in diameter. Therefore, we also considered a scenario with an enlarged vent diameter.

### Modelling of PDC dynamics and hazard

During collapse regimes, the eruptive mixture at the time of collapse can be relatively dilute, especially in oscillating columns where it can have an average density as low as ~ 10 kg/m^3^, i.e. a particle volume concentration of less than ~ 10^−2^ (Wilson et al. 1980; Woods 1988; Neri and Dobran 1994; Esposti Ongaro et al. 2002, 2008a; Suzuki and Koyaguchi 2012; Trolese et al. 2019). Nonetheless, PDCs manifest a steep vertical stratification in the proximal region around the vent, where *breccias* are often observed (Branney and Kokelaar 2002; Valentine and Sweeney 2018). Such PDCs can be described as a basal, concentrated layer overlain by an upper, more dilute (stratified) and more mobile ash cloud (cf. Doyle et al. 2010).

A common approach to studying the subsequent PDC dynamics and their hazard is to adopt homogeneous mixture, depth-averaged models, which have the advantage of a fast numerical solution (e.g. Patra et al. 2005; Shimizu et al. 2017; de’ Michieli Vitturi et al. 2019). However, single-layer models always impose a dichotomy (and the need for a choice) between dominantly frictional (concentrated) or dominantly inertial (dilute) PDCs. In this regard, inertial flow models (Sparks 1976; Sparks et al. 1978; Bursik and Woods 1996; Dade and Huppert 1996) describe PDCs as relatively dilute (particle concentration < 10^−2^), turbulent gas-particle flows, which lose mass, increase in buoyancy, and eventually stop their horizontal motion, lifting off as a consequence of particle settling and air entrainment. Such models are more suited to modelling PDCs in the absence of a significant topographic slope or for very long runouts such as those associated with high aspect ratio ignimbrites. In such cases, the basal, concentrated layer acts as a *depositional system* and does not control PDC dynamics (the *transport system*; Fisher et al. 1993; Giordano and Doronzo 2017). Such an approach has also been applied to calderas, where the average volcanic slope is negligible (Brand et al. 2014; Neri et al. 2015a, b; Esposti Ongaro et al. 2016). At the other end-member, frictional flow models (Patra et al. 2005; Kelfoun et al. 2009; Doyle et al. 2008; Roche et al. 2011) describe PDCs as concentrated granular flows (with particle volume concentrations of > 10^−1^), controlled by frictional forces. They are more suited to low aspect ratio PDCs, often confined to the volcano flanks, or to describe the behaviour of the basal part of a stratified PDC. Most of the difficulties in the physical description of PDCs is related to the interplay between these two end-members in natural (Druitt et al. 2002b; Ogburn et al. 2014; Bernard et al. 2014; Capra et al. 2016) and laboratory PDCs (Breard and Lube 2017), where the multiphase nature of the mixture also poses significant physical and mathematical challenges (Pitman and Le 2005; Pudasaini and Mergili 2019). An attractive and promising alternative to single-layer models is provided by two-layer depth-averaged models (Doyle et al. 2010; Kelfoun 2017; Shimizu et al. 2019; Gueugneau et al. 2019), in which PDC stratification is simplified into a concentrated, basal layer underlying a dilute ash cloud. However, in these models, it can be challenging to calibrate *a priori* empirical mass, momentum, and energy exchanges between the two layers. This adds to the difficulty of calibrating rheological models for both the concentrated and turbulent layers and to properly set the source conditions for column collapse.

Here, we use the three-dimensional, multiphase flow model PDAC (i.e. Pyroclastic Dispersal Analysis Code; Neri et al. 2003; Esposti Ongaro et al. 2007; Carcano et al. 2013) to numerically simulate the development, instability, and collapse of a subplinian eruption column and the generation and propagation of PDCs over the topography around SDG. All model equations and the main underlying assumptions are summarized in Appendix 1. The advantage of using non-equilibrium multiphase flow models is that they offer a comprehensive description of stratified PDCs (Esposti Ongaro et al. 2008b, 2012; Esposti Ongaro et al. 2016; Dufek and Bergantz 2007b; Dufek 2015; Benage et al. 2016). In particular, 3D models can describe PDC proximal stratification, formation of the basal layer by particle settling, and generation of an overlying ash cloud due to shear flow mechanisms.

The reliability of the PDAC model in describing the main large-scale behaviour of volcanic plumes, for the range of mass eruption rates apparent here, has been demonstrated by a 3D plume model inter-comparison study (Costa et al. 2016; Suzuki et al. 2016; Esposti Ongaro and Cerminara 2016). However, a quantitative, rigorous evaluation of model-related uncertainty on a full eruption scenario involving plume formation, instability, and PDC generation is not yet possible. This is a more general issue, related to the validation of numerical models in volcanology (Oreskes et al. 1994; Esposti Ongaro et al. 2020). We thus base our discussion on the relatively large number of 3D numerical simulations performed in this study, with input conditions derived from field work carried out at SDG and published in Boudon et al. (2008), Komorowski et al. (2008, 2012, 2013), and Legendre (2012). In evaluating the reliability of our results and the potential effect of the adopted numerical approximations on the model output, we also rely on our 2D/3D numerical simulations at Vesuvius (Esposti Ongaro et al. 2002, 2008a; Neri et al. 2007), Soufrière Hills, Montserrat (Esposti Ongaro et al. 2008b), Campi Flegrei (Todesco et al. 2006; Esposti Ongaro et al. 2010), Mount St. Helens (Esposti Ongaro et al. 2012), and on similar modelling works by Dufek and Bergantz (2007a, b) and Benage et al. (2016).

## Simulation assumptions and source parameters

Our modelling assumes a sustained event, i.e. stationary conditions at the vent producing a collapsing column. In Appendix 2, we also discuss the application of the method to a single, impulsive explosion. Steady-state boundary conditions are imposed at the vent, coinciding with the exit section of the crater. We initially assume an average mass flow rate of 7 × 10^6^ kg s^−1^ ejected from a circular vent located on the present summit of the SDG dome, as based on Komorowski et al. (2008). Initial temperature was set to 1050 K (777 °C) and water content to 2 wt.%, resulting in a mixture density of around 12 kg/m^3^. Although the water content is lower than the 5 wt.% estimated from petrological analysis of the erupted materials (Boudon et al. 2008; Pichavant et al. 2018), our previous studies (Esposti Ongaro et al. 2008a) show that a value of 2 wt. % is the upper threshold for which subplinian eruption plumes with mass flow rates and temperatures in the investigated range can collapse and produce PDCs. It is also worth recalling that reduction of volatile content in the gas-pyroclast eruptive mixture is possible by many mechanisms, including gas entrapment in pumice (Kaminski and Jaupart 1998) and permeable degassing (La Spina et al. 2017).

The granulometry of juvenile particles was derived from data given in Komorowski et al. (2008) by adopting three particle classes with diameters of 1000 μm (50 wt.%), 250 μm (24 wt.%), and 30 μm (26 wt.%), and densities of 1200, 2000, and 2,600 kg/m^3^, respectively. Although this granulometry is finer than the actual subaerial deposit of the 1530 CE eruption of SDG, it represents a compromise between the need to account for a relatively coarse component of the pyroclastic phase and the capability of our numerical model to treat coarse-grained phases. Moreover, the choice of a finer granulometry is justified by the fact that a large part of the material produced in subplinian eruptions is fine-grained and deposited distally (Sparks and Walker 1977; Marti et al. 2016), in our case in the sea where it is not easily accessible and thus cannot be included in estimates of the total grain size distribution. The three particulate phases are initially in mechanical and thermal equilibrium with the gas, but they are characterized by different degrees of coupling with the carrier fluid flow, so that non-equilibrium phenomena (between gas and particles and between different particles) developing during the eruption can be analysed with the model. Input parameters for grain size distribution are given in Table 1.

**Table 1:** Properties and mass/volume fractions of solid particle phases (named P1, P2, and P3) used to represent the input grain size distribution for numerical simulations SP1–SP4 in Table 2. Grain size data are taken from Komorowski et al. (2008)

Phase	Gas	P1	P2	P3
Diameter [μm]	n.a.	1000	200	50
Density [kg/m^3^]	0.21	1200	2000	2600
Bulk density [kg/m^3^]	0.21	6.0	3.0	3.12
Mass fraction [wt. %]	1.7	48.7	24.3	25.3
Relative solid mass fraction	n.a.	49.5	24.8	25.7
Volume fraction	0.9923	0.0050	0.0015	0.0012

Four scenarios have been selected, named SP1 through SP4, whose main input parameters are given in Table 2. Input parameters have been set to cover the estimated range of mass eruption rate and water content. A list of simulations performed to assess the robustness and sensitivity of the results to the numerical discretization and boundary conditions is given in Appendix 1.

**Table 2:** Input parameters for the four simulated scenarios

	SP1	SP2	SP3	SP4
**Input**				
*Inlet radius* [m]	38	45	52	104
*Inlet velocity* [m/s]	127	90	70	70
*Gas pressure* [Pa]	10^5^	10^5^	10^5^	10^5^
*Mixture density* [kg/m^3^]	12	12	12	12
*Mixture temperature* [K]	1050	1050	1050	1050
*Water content* [wt. *%*]	2	2	2	2
*Mass flow rate* [kg/s]	7 × 10^6^	7 × 10^6^	7 × 10^6^	2.8 × 10^7^
**Output**				
*Estimated percentage of collapse* (± 10%)	50%	70%	90%	90%

Simulation SP1 has a vent radius of 38 m and an exit velocity of 127 m/s. It was run over a 6 × 6 km^2^ digital elevation model centred on the summit area with uniform horizontal resolution of 10 m. We used a non-uniform rectilinear 3D computational grid with horizontal resolution of 10 m in the area around the vent and 50 m at the North, South, East, and West boundaries, and 20 m vertical grid size up to 1500 m a.sl., increasing up to 200 m at the top of the domain (12 km). The position of the vent was placed at [642985; 1774280] in UTM 20N WGS84 Cartesian projection. Simulation SP2 has the same mass eruption rate and mixture density as SP1; however, the vent radius is 16% larger (an increase from 38 to 45 m). As a consequence, exit velocity is reduced to 90 m/s. The domain is extended to 11 × 11 km^2^, with the same resolution (10 m) at the vent. Simulation SP3 also has the same mass eruption rate of SP1; however, the vent radius is 37% larger (52 m). As a consequence, exit velocity is further reduced to 70 m/s. The computational domain is extended to 20 × 12 km^2^ in the horizontal directions, and the horizontal cell size around the vent is 20 m to keep the computational cost manageable. However, we made simulations at 10 m on a reduced domain to ensure that the main features are captured at the lower resolution (see Table 3). Finally, simulation SP4 has an increased mass eruption rate, four times larger than SP3 (i.e. 2.8 × 10^7^ kg/s), with a vent radius twice as large (104 m), but with the same exit velocity and the same eruptive source parameters. Such a scenario might represent the final summit crater collapse stage during a subplinian event and should be considered as an extreme but credible scenario for the culminating phase of the eruption.

Occurrence of partial edifice collapse at the onset of, or during, a magmatic eruption can have profound consequences on the morphology of the vent that can lead to its enlargement and, thus, promoting full collapse of the eruptive column (as first demonstrated with a mathematical model by Wilson et al. 1980). Indeed, shortly after the onset of the 1530 CE eruption, the edifice partially collapsed to the South leading to the emplacement of an 80 ± 40 × 10^6^ m^3^ debris avalanche deposit that reached the sea and left a large ca. 500-m-wide horseshoe-shaped crater open to the South and South-West (Boudon et al. 2008; Fig. 3). Komorowski et al. (2005) and Legendre (2012) have shown that eight out of the nine edifice collapse events that occurred in the last 9150 years at SDG were associated with magmatic eruptions, and four of them (50%) were associated with major explosive phases (subplinian or plinian). Increase of the mass flow rate by a factor of four in simulation SP4 can represent the effects produced by an asymmetric collapse, focussing the whole pyroclastic flow mass to one specific quadrant. We show in Appendix 3 that the resulting maps of PDC invasion are comparable when the same mass flow per unit of angle is considered. Such asymmetric subplinian column collapse has been shown to have favoured the formation of highly mobile although low-volume pumiceous pyroclastic flows in one main direction during the 2010 multiphase eruption of Merapi volcano (Komorowski et al. 2015). Clearly, such focussing of the mass in one or a few river valleys favours efficient mobility and longer runout than expected in the case of such small-volume PDCs.

To describe the column regime, we adopt the same approach used for Vesuvius by Esposti Ongaro et al. (2008a) and by Trolese et al. (2019), where the transition from the fully convective to fully collapsing regimes was quantitatively characterized by the percentage of collapsing mass (i.e. the ratio between the maximum mass collapse rate and the mass eruption rate at the vent). These studies, along with Esposti Ongaro et al. (2016), provided evidence that the percentage of collapse is the most important parameter controlling PDC propagation.

## Numerical simulation results

### Partial collapse scenarios

**Fig. 4:**
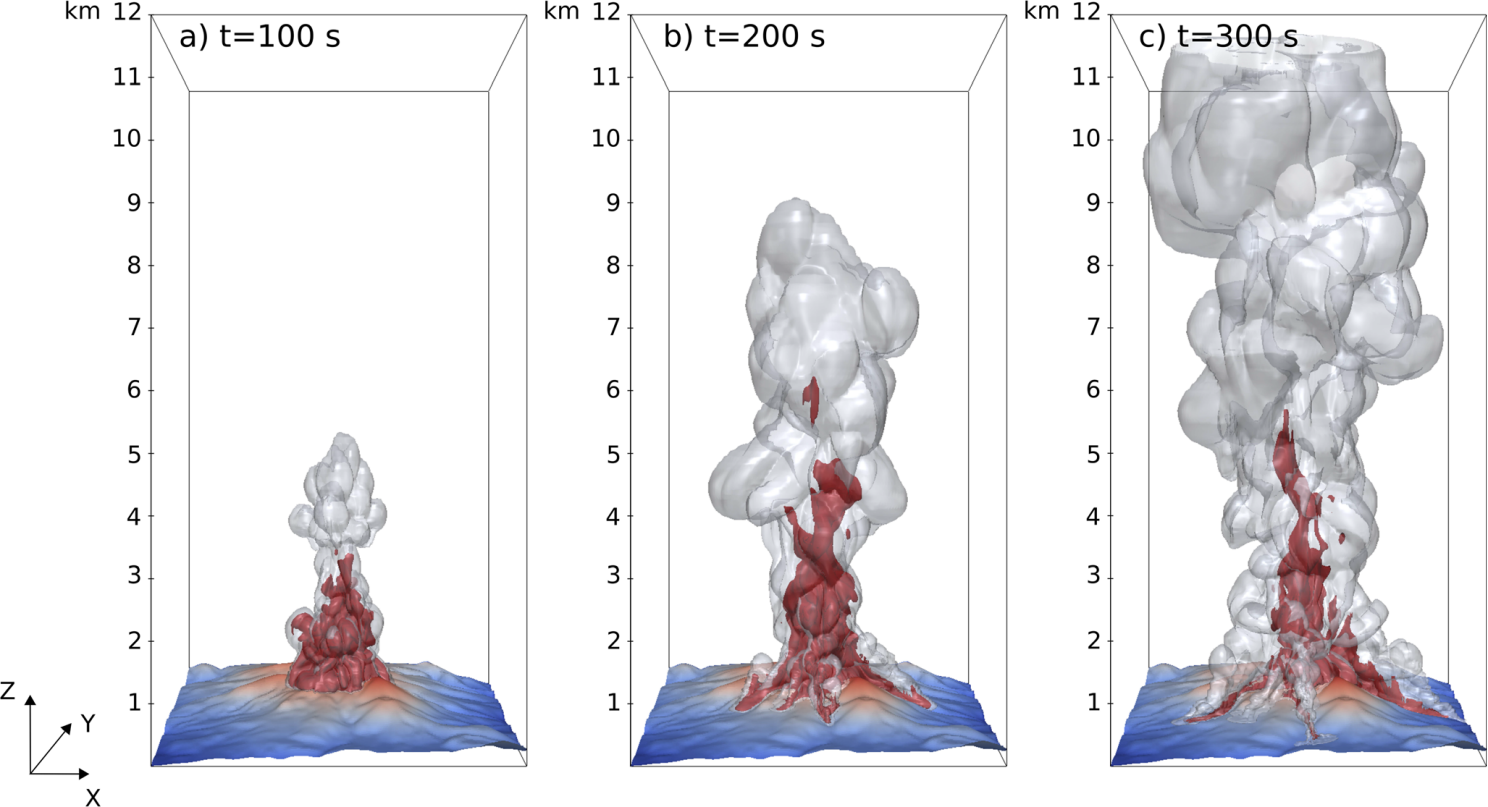
Simulation of a subplinian eruption column with 50% of collapse and formation of channelized PDCs on the 3D topography (run SP1, Table 1). **a** *t* = 100 s, **b** *t* = 200 s, and **c** *t* = 300 s, from the beginning of the collapse phase. Coloured zones are isosurfaces that represent fine ash (50 μm diameter) volume concentrations of 10^−5^ (red, internal) and 10^−7^ (grey, transparent external). The *X*-axis is oriented West-East, and *Y* is oriented South-North

Numerical simulations describe in 3D the formation of the volcanic jet, its instability and partial collapse, resulting in the simultaneous formation of a sustained plume and PDCs. In scenario SP1 (Fig. 4), partial collapse starts at about 35 s after the onset of the subplinian eruptive phase, while the collapsing portion of the plume reaches the foot of the eruptive column at about 50 s. At 100 s (Fig. 4a), incipient PDCs are still confined within the summit area while the central convective plume develops. At 200 s (Fig. 4b), the turbulent, convective part of the plume has reached a height of about 9 km above the vent, while PDCs start to propagate radially into valleys. At 300 s (final simulation time; Fig. 4c), the plume has reached the top of the domain at 12 km, while PDCs have almost stopped in lateral valleys. Fine ash elutriated from PDCs contributes to the formation of proximal co-ignimbrite plumes that merge with the main central plume. Isosurfaces of particle concentration (Fig. 4c) highlight the role of the near-vent topography in controlling the PDC propagation. In particular, PDCs are not able to overcome the topographic barriers on the North-West side of the summit, so that these flows are diverted into South-West valleys, along the *Nez Cassé* ridge (Fig. 2). Analogously, the steep valley to the South is the main collector of all PDC developing towards South and Southeast, where fast-moving (10–15 m/s) currents form. The most concentrated PDC propagates along the Eastern valley.

**Fig. 5:**
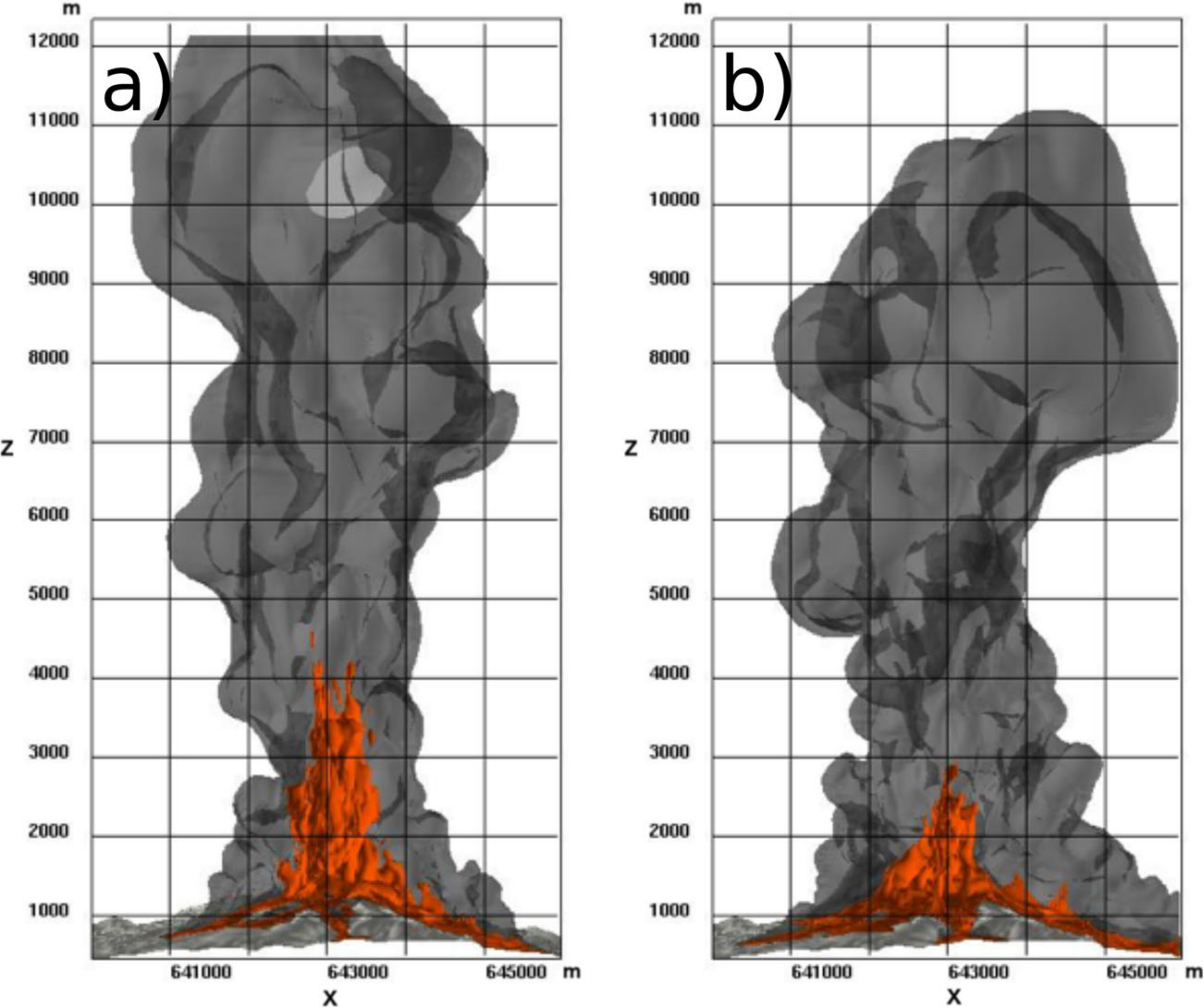
Comparison between 3D numerical simulations of the partial collapse of an eruptive column in a subplinian scenario with different collapse percentages. **a** Run SP1, 50% collapse. **b** Run SP2, 70% collapse. Isosurfaces represent total particle concentration in the atmosphere of 10^−4^ (inner, orange) and 10^−6^ (outer, grey), 280 s after the beginning of the simulation. The horizontal *X*-axis gives the UTM (West-East) coordinates, *Z* is the elevation above sea level, in metres

In scenario SP2, characterized by a lower exit velocity and larger vent radius, the height of the momentum-driven jet decreases due to the lower velocity, leading to a lower collapse height. In Fig. 5a, b, this is represented by the isosurface of 10^−4^ particle concentration, whose maximum elevation decreases from about 2500 to 1500 m above the summit (from 4000 to 3000 m above the sea level). The computed percentage of collapse is about 70%, which is still in the regime of an oscillating column. Pyroclastic density currents are mostly directed to the South-West, South, and Eastern sectors, but their runout after 300 s is limited to the proximal region (within < 2–3 km of the vent). At this point, PDCs have almost completely stopped moving horizontally and revert to buoyant clouds. At this stage, air entrainment is very effective in diluting the PDC and lowering its temperature. Although up to 70% of the mass is collapsing, a large fraction is rapidly elutriated by proximal co-ignimbrite plumes developing as soon as the collapsing portion of the plume impacts the ground. This occurs all around the vent, so that only about 20% of the total erupted mass feeds PDCs.

### Full collapse scenario

**Fig. 6:**
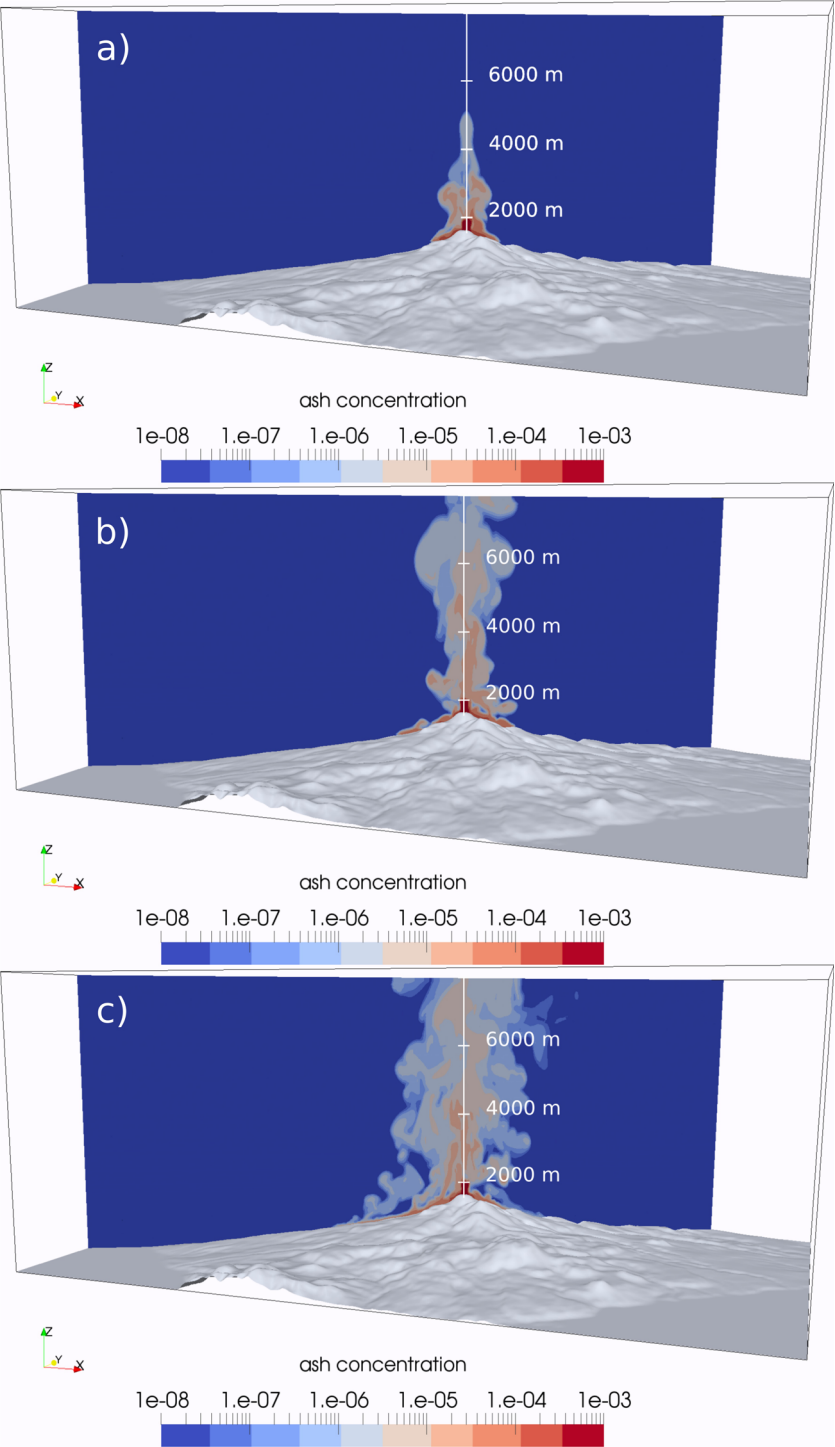
3D sequence of fully collapsing (> 90%) subplinian eruption (run SP3) at **a** *t* = 100 s, **b** *t* = 200 s, and **c** *t* = 380 s, after the beginning of the collapse phase. The colour scale represents the volume concentration of the fine ash (diameter 50 μm) on a logarithmic scale

The input conditions of SP3 produce a regime of almost total, stationary collapse (about 90% of the mass collapsing). The height of the jet is further lowered to about 500 m above the summit, and the structure of the eruptive column is significantly different, with a pyroclastic fountain above the vent that isotropically feeds PDCs, which spread radially all around the vent (Fig. 6). As observed at Vesuvius by Esposti Ongaro et al. (2008a) and by Trolese et al. (2019), this regime feeds more concentrated and more mobile PDCs, which form stratified currents with higher basal particle concentration, larger inertia, and greater runout. Even in this case, however, PDCs are not able to overcome the topographic obstacles on the Northern side of the flank collapse structure or major scarps around the summit and are mostly focussed (by topography) towards the South, West-Southwest, and East.

**Fig. 7:**
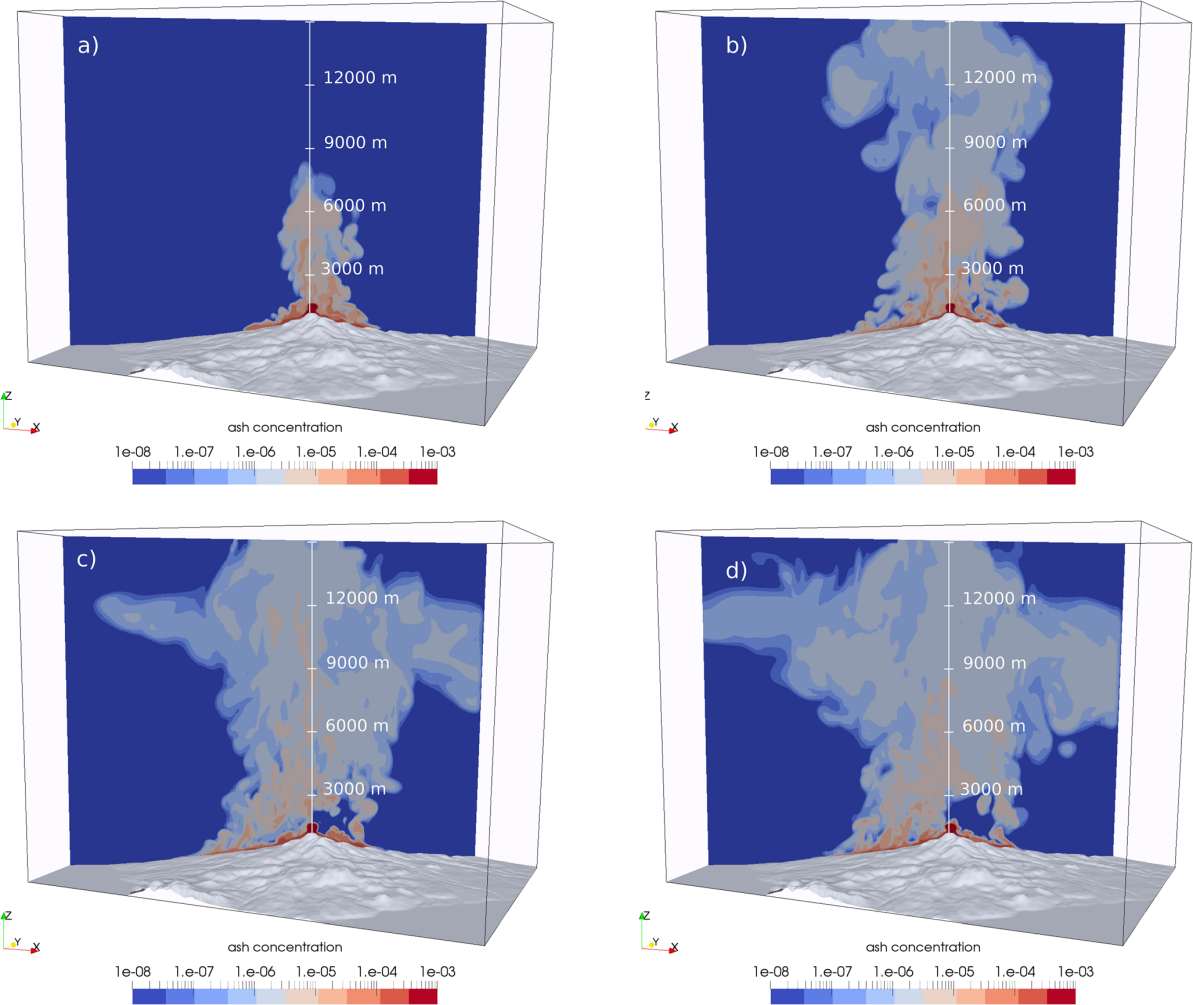
3D sequence of full (> 90%) collapse, with increased mass eruption rate of about 3 × 10^7^ kg/s (run SP4) at **a** *t* = 200 s, **b** *t* = 400 s, **c** *t* = 600 s, and **d** *t* = 800 s after the beginning of the collapse phase. The colour scale represents the volume concentration of the fine ash (diameter 50 μm) on a logarithmic scale

Simulation SP4 (Fig. 7) also displays a regime of almost total collapse (larger than 90%). Because of the increased mass flow rate, PDCs are of higher velocity and concentration and have a much longer runout. To the South-West, the collapsed mass is deflected by the *Nez Cassé* ridge and move down the *Savane à Mulets* area towards the town of *Saint-Claude* (Fig. 2). Pyroclastic density currents initially branch and follow two main directions (one main stream in the *Rivière Noire* valley and a second, minor stream through the *Bains-Jaunes* area; Fig. 2), but they eventually merge after about 2 km. About 300 s after the beginning of the collapse phase, PDCs have reached the inhabited region of *St. Claude* and become branched along the main valleys. At the same time, a large part of the collapsed mass is conveyed along the *Rivière du Galion* valley to the South into a narrow valley section (the *Chute du Galion*; Fig. 2), and branches into two main flows. The first eventually merges South of the town of *Saint-Claude* (*Rivière Dugommier* valley) with the South-West branch, while the second forms a channelized PDC propagating 6–7 km South from the vent, overtopping the *Palmiste* ridge to flow down towards the town of *Trois-Rivières*, along the *Rivière du Petit-Carbet* valley (Fig. 2). To the East, the main branch is directed along the *Rivière du Carbet* (*Grand Carbet*) valley, towards the headland of *Pointe-du-Carbet* (Fig. 2), where it is able to reach the sea (about 10 km from the vent). At the coast, the flow still possesses a significant dynamic pressure (> 1 kPa). A minor branch is channelized along the *Rivière du Perou*, but extends no more than 3 km.

### PDC invasion maps

Maps of PDC invasion were plotted by interpolating the 3D numerical results on isosurfaces at constant height above the topography. We take the first cell above the topography as representative of ground-level PDC conditions. Ground-level values are thus average values for the first 20 m above the topography (10 m for fine mesh simulations; Appendix 1). Such an averaging is implicit in the adopted finite volume computational technique and numerical grid. We use temperature and dynamic pressure (i.e. the kinetic energy per unit of volume) as the two most significant variables for hazard assessment (Esposti Ongaro et al. 2002; Gurioli et al. 2005). Dynamic pressure is calculated as ${P}_{\mathrm{dyn}}=\frac{1}{2}{\rho}_m{\left|{\boldsymbol{v}}_m\right|}^2$ , where *ρ*_*m*_ and |***v***_*m*_|^2^ are the mixture density and magnitude of the velocity in the first computational cell above the topography. Maps of temperature are shown at the final simulation step (i.e. after 300 s for SP1, 380 s for SP2, 550 s for SP3, and 800 s for SP4). This is the time at which the most concentrated (basal) part of the current stops to advance. Following past simulation experiments and comparisons with real PDC events (e.g. Esposti Ongaro et al. 2008b, 2012) suggests that this is the best estimate of the actual PDC runout, even though the dilute cloud is still capable of slow advance, especially in the absence of wind and atmospheric turbulence in the model description. For dynamic pressure, we plot the maximum value reached at each grid point during the simulation.

**Fig. 8:**
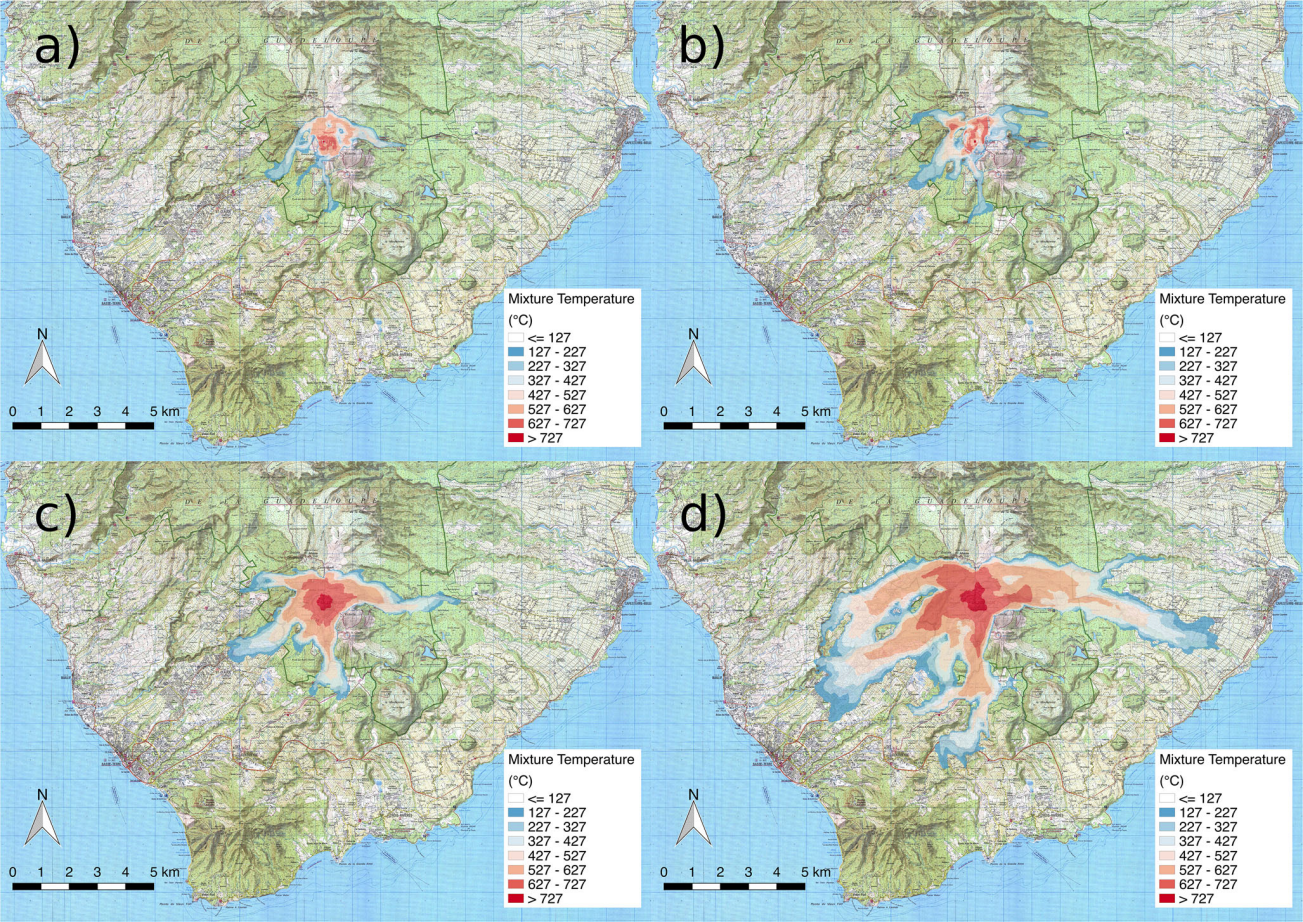
Final maps of mixture temperature superposed to the IGN cartography, showing the inhabited regions around the volcano. Maps are given for simulations **a** SP1, **b** SP2, **c** SP3, and **d** SP4

Maps of mixture temperature at 10 m above the topography are given in Fig. 8a–d for simulations SP1, SP2, SP3, and SP4. Partial collapse events (SP1 and SP2) have very similar distributions (Fig. 8a,b), with a more pronounced branch to the North-West in SP2. Both scenarios are characterized by oscillating columns with very efficient air entrainment and flow cooling, generating PDCs that are quickly stopped by air drag and lift-off. There are no concentrated PDCs beyond a distance of about 2.5 km to the South and South-West, and after about 3 km to the East-South-East. Their impact on inhabited regions is consequently quite limited. Full collapse, boiling over scenarios SP3 and SP4, on the contrary, are able to spread PDCs over the entire South-West sector and towards the East (Fig. 8c,d), with some topographic channelling and proximal morphological control. The PDCs generated by boiling over are able to maintain their initial high temperature (above 700 K, or 427 °C) in the basal, more concentrated part of the flow. The only sectors preserved from PDC invasion are towards the South-East, a result of the sheltering effect of the *Echelle-Citerne* complex, and to the North, which is protected by *Carmichael* and *Grande Découverte* edifices*.*

**Fig. 9:**
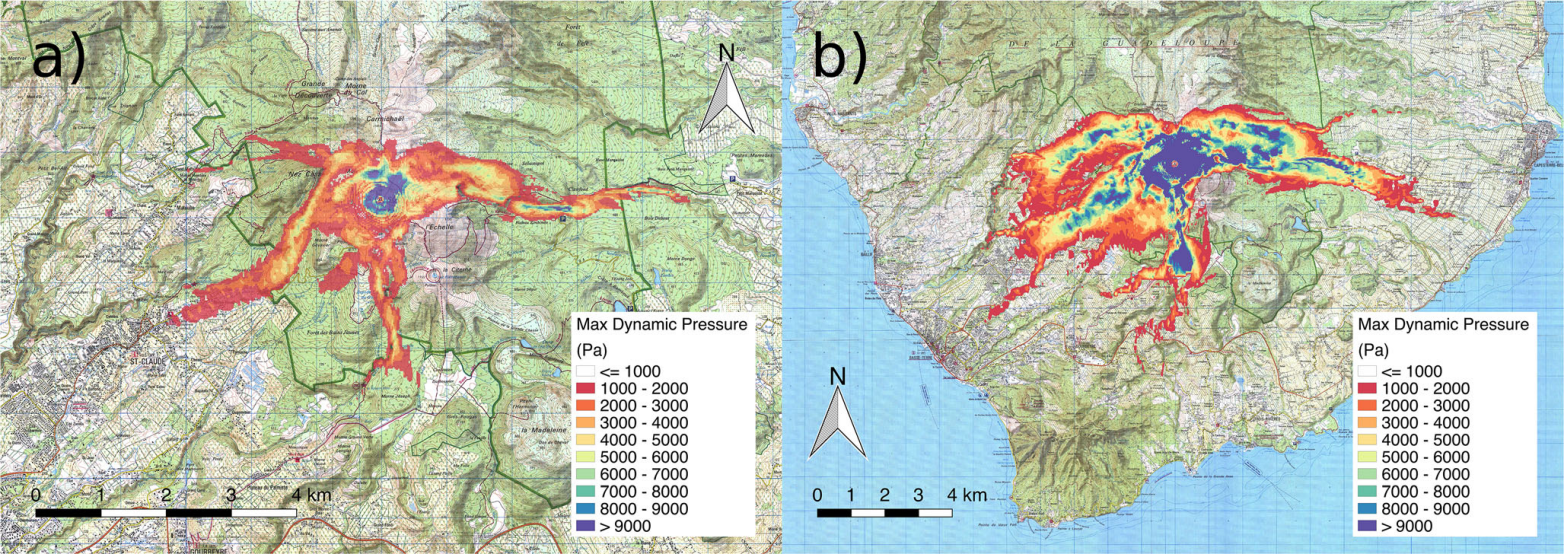
Maps of maximum dynamic pressure estimated for each point in the domain for scenarios **a** SP3 and **b** SP4

For simulations SP1 and SP2, dynamic pressure maps do not show values of greater than 1 kPa outside of the summit area and the low values recorded in the simulation (mostly associated with velocity of atmospheric air) make the maps extremely noisy, so that we do not present the maps here as they are meaningless. As a reference, a dynamic pressure of 1 kPa is sufficient to break windows, whereas at 10 kPa failure of reinforced masonry can be expected (Jenkins et al. 2010, 2013a). Figure 9 represents the maximum dynamic pressure for runs SP3 and SP4, with values lower than 1 kPa filtered out. For SP3, dynamic pressures above 10 kPa are estimated only close to the summit and along the *Rivière du Carbet* valley, whereas in the other valleys and in the proximal region (within 2–3 km from the vent) values up to 5 kPa can be expected (Fig. 9). Dynamic pressures of up to 3 kPa are estimated in the more distal (> 4 km) and inhabited regions, including in the town of *St. Claude.* For SP4, the full collapse regime and the increased mass flow rate make the area of significant impact much wider (cf. Fig. 9a, b). To the West, *St. Claude* and *Matouba* would be subject to PDCs with dynamic pressures exceeding 10 kPa at the edge of the town closest to the vent and > 5 kPa over most of the town (Fig. 9b). Pyroclastic density currents with dynamic pressures of > 1 kPa would extend as far as the town of *Basse-Terre*, and to the South, dynamic pressures of > 1 kPa is predicted at distances out to about 6 km. To the East, both the *Rivière du Carbet* and *Rivière du Perou* valleys would be affected, with the inhabited region of the town of *Capesterre-Belle-Eau* impacted heavily by dynamic pressures of > 3 kPa. The region affected by dynamic pressures of > 1 kPa extends almost to the coast down the *Rivière du Carbet* valley (Fig. 2).

### Control of proximal morphology on PDC distribution

**Fig. 10:**
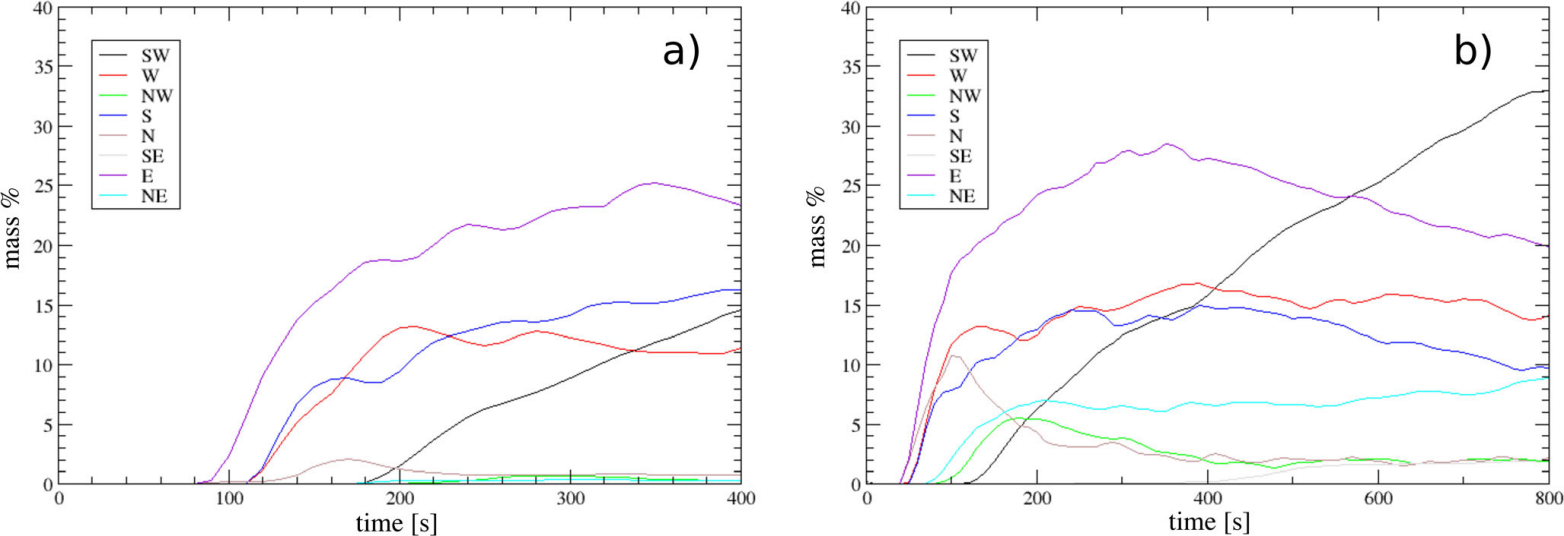
Ratio of mass conveyed into valleys by PDC with respect to the collapsed total mass for simulations **a** SP3 and **b** SP4

The distribution of PDCs around the vent is controlled by the morphology of the volcano summit (Fig. 2b). In our simulations, we impose a circular vent and homogeneous conditions with no wind, so that partial and total collapses intermittently feed PDCs uniformly around the vent Pyroclastic density currents are initially confined by the walls of the flank collapse structure, but the progressive superposition of multiple collapsing events then favours the propagation of PDCs beyond this limit. In Fig. 10, we show the temporal evolution of the mass conveyed in the different sectors of the volcano for simulations SP3 and SP4.

The main topographic obstacle in the Northern sector is represented by *Carmichael* and *Grande Découverte* edifice in the North-West sector and the *Montagne de la Capesterre* edifice in the North-East sector (Fig. 2), which in our simulations prevents the propagation of PDCs to the North (only about 2% of the collapsing mass is channelized along the *Rivière St Louis* valley in simulation SP4). However, part of the collapsed mass (11% and 15% of the collapsing mass in scenarios SP3 and SP4, respectively) is able to surmount the first topographic barrier and is channelized to the West towards the town of *Matouba*. Towards the East, PDCs are confined by the *Echelle* edifice and are channelized along the *Rivière du Carbet* valley, where 20–25% of the collapsing mass is conveyed in both SP3 and SP4. Part of the mass (about 9%) is channelized to the North-East in the *Rivière du Perou* valley in simulation SP4. Toward the South, in both SP3 and SP4, between 10 and 15% of the collapsed mass is conveyed along a narrow ravine through the *Chute du Galion* to branch downstream into two main streams, one being channelized along the *Rivière du Galion* valley and the other more diffused around the *Morne-Joseph (Rivière de la Grande Anse*; Fig. 2).

In both SP3 and SP4, most of the collapsing mass is deflected to the South-West by the *Nez Cassé* ridge and down the *Savane à Mulets – Plateau Dimba* area towards the town of *Saint-Claude*. In this direction, this appears to be the main “outlet” of the summit morphological structure. Pyroclastic density currents initially branch into two main valleys: the *Rivière Noire* (West sector) and the *Rivière Dugommier* (South-West sector), but they merge downstream after about 2 km. In contrast to the other sectors, where an equilibrium is reached between sedimentation and elutriation (so that the mass ratio becomes more or less constant), the mass ratio increases down-flow in the South-West sector, denoting an accumulation of the deposited material but also reflecting the increasing contributions from the Southern and North-Western PDC branches which merge down-flow. The remaining collapsing mass remains within the summit area (35% for SP3, 10% for SP4).

The topography of the SDG massif, the likelihood of partial edifice collapse, and the high-water content of the magma all favour an almost total collapse of the eruption column and a strong control of topography on the distribution of PDCs. These factors have been shown to be a very efficient means of enhancing the mobility of PDCs during the 2010 eruption of Merapi (Komorowski et al. 2015). At SDG, focussing of PDC material down ravines and deep canyons for several kilometres is likely to contribute to hotter than expected gas-rich PDCs, with greater potential impacts on humans and the built environment. Moreover, focussing more mass at greater distances will increase the likelihood of secondary turbulent PDCs being generated that could overtop topographic divides and barriers and invade areas not directly exposed to the channelized and concentrated parental PDCs. This has been shown to have been the case during the Soufrière Hills eruption of 1997 (Druitt et al. 2002b) and at Merapi volcano in 2010 (Komorowski et al. 2015). Deposition of PDC material in valleys will also favour the generation of debris flows and lahars after the end of the eruption (cf. Scott et al. 1996). Our modelled PDCs from total column collapse scenarios are able to reach the coastline at least to the East, entering the sea close to the town of *Capesterre-Belle-Eau* and to the South-West in the town of *Basse-Terre* (the location of the Guadeloupe préfecture, i.e. the centre of decision making and governance by the authority representing the national government). Depending on the total mass and the mass flux into the sea, such PDC entry into the sea could lead to the generation of tsunamis (Begét 2000; Tinti et al. 2003; Paris 2015) that could refract along the coastline North and South from the entry point as well as propagate eastward towards the islands of *Les Saintes* and *Marie-Galante* (Fig. 2). Such cascading phenomena must be further modelled and their potential impact quantified in adequate simulations.

## Discussion

Numerical results describe the spatial (in 3D) and temporal detail of formation, instability, and partial collapse of eruptive columns. For the case considered here, the percentage of collapse, at constant mass eruption rate, increases with the vent radius. Partial collapse episodes generate pyroclastic accumulation at the base of the plume which then flows away from the base as PDCs. The horseshoe-shaped collapse structure of the summit area and the incised nature of the volcano flanks then control the areal distribution of the PDCs.

### PDC mobility and intensity

Simulations show that partial collapses occurring from an oscillating column intermittently generate axisymmetric spreading of PDCs across all sectors of the volcano. However, PDC intensity (i.e. their mass flow rate per unit of angle) is limited, even at a collapse rate of about 70%. Our modelled PDCs are significantly cooled and slowed by air entrainment, gas drag, and lift-off. Such an observation is supported by numerical modelling by Trolese et al. (2019) who showed that PDC temperature is linearly correlated with the percentage of column collapse.

Our numerical simulations show that PDCs from oscillating columns are unable to significantly impact the inhabited zones around the volcano. This, however, is incompatible with the sedimentological observations of PDC deposits in the town of St. Claude and in the main drainages, at distances of 6–7 km (Boudon et al. 2008; Fig. 3). To achieve such distances, our modelling shows that a regime of full collapse (i.e. >90% of collapse) and low fountaining would be necessary, with an increased mass flow rate of 2.8 × 10^7^ kg/s. Pyroclastic density currents generated in a regime of full column collapse are able to reach distances of > 8 km from the vent in less than 15 min, and to overtop topographic barriers that otherwise block flows. Such PDCs are able to reach distal areas while maintaining dynamic pressures of up to 3–5 kPa and temperatures of up to 500 K (or 230 °C) in the most onland proximal locations at distances of 5–6 km, which is well into the inhabited region.

### Comparison with the reference scenario

The value of 2.8 × 10^7^ kg/s required to impact inhabited zones is to the upper bound of the mass flow rate for the 1530 CE eruption at SDG. It is worth noting that reconstructions of the mass eruption rate based on an estimate of plume height do not consider that, during the collapse phase, the mass feeding the plume is significantly reduced and, thus, the maximum eruption rate is likely underestimated (potentially by a factor of 10 in the case of 90% collapse; Trolese et al. 2019). Moreover, regimes of > 90% collapse are likely favoured by potential partial edifice collapse. The partial collapse of the edifice that occurred shortly after the onset of the 1530 CE eruption, which left a large ca. 600–800-m-wide and ca. 1.2-km-long horseshoe-shaped crater open to the South and South-West (Boudon et al. 2008; Fig. 3), is much larger than the vent radius of 104 m that we assumed for SP4. This greater vent diameter is therefore likely to have had a profound effect on the dynamics of the eruption column and explains why PDCs during the 1530 CE eruption were able to overcome topographic obstacles on the Northern side of SDG and flow into the upper part of the *Rivière St. Louis* valley, reaching a distance of about 5–6 km as confirmed by field mapping (Fig. 3; Boudon et al. 2008; Komorowski et al. 2008). This is in line with observations during the 2010 eruption of Merapi, where the presence of a large 400 × 300-m-wide and 150–200-m-deep horseshoe-shaped crater created by the initial phases of the eruption strongly controlled the direction of full collapse of the column in the final phase of the eruption, hence favouring the excessive runout of very small-volume yet mobile pyroclastic flows that travelled about 15.5 km from the vent (Komorowski et al. 2013).

### Uncertainty on model results

Many aspects of PDC dynamics remain difficult to investigate, because of the lack of knowledge on the constitutive properties of gas-particle mixtures and the spatial resolution of the numerical simulations. In particular, it is still challenging to describe the rheology of concentrated granular mixtures in 3D (Breard et al. 2019) and to correctly reproduce vertical stratification when the vertical grid size is comparable to or larger than the thickness of the basal layer. Our model-based estimates of PDC runout, temperature, and dynamic pressure should then be taken as relative measures and are intended as average values over the first 10–20 m above the topography. In previous studies with comparable numerical parameters, we were able to estimate that the maximum values of dynamic pressure can be up to a factor ~ 5 larger as a consequence of density stratification (Esposti Ongaro et al. 2008b). Grid resolution might also affect the description of PDC deflation and pressurization at the impact zone (Valentine and Sweeney 2018). The binning of the grain size distribution can also influence the results of simulations. Although Neri et al. (2003) and Carcano et al. (2014) showed that three classes are a minimum number of bins required to achieve a satisfactory description of multiparticle dynamics, the uncertainty associated with incomplete description of the distribution is still to be evaluated (Esposti Ongaro et al. 2020). Further uncertainty in simulation results are associated with physical aspects of numerical modelling, such as sub-grid scale turbulence models and ground boundary conditions. The issue of ground boundary conditions, in particular, is strictly related to the vertical adopted numerical grid size. As already observed by Esposti Ongaro et al. (2008b, 2012), coarse grids do not allow the accurate resolution of the stratification of the flow and interaction with the lower, rough, topographic boundary. In particular, at least five cells are required to describe the flow boundary zone. This is not the case in most of our numerical simulations for the distal part of the PDC runout, where the uncertainty on numerical model results is therefore larger than in the proximal region. However, in our experience, the adopted vertical grid size is a good compromise to describe the main large-scale PDC features.

Finally, we have shown that the mass flux of pyroclastic material conveyed in PDCs is the main parameter controlling their mobility (invaded area and runout) and, consequently, their impact. We stress here that column collapse height is almost irrelevant in the determination of PDC runout and intensity, in the case of partial collapse dynamics. This observation is consistent with results of integral inertial models (Dade and Huppert 1996; Esposti Ongaro et al. 2016) but is contrary to empirical models based on the energy-line approach (e.g. Sheridan and Malin 1983; Tierz et al. 2016; Sandri et al. 2018). Although we present a relatively limited set of eruptive conditions (e.g. vent velocity, density, temperature, grain size), the same behaviour has been observed by Esposti Ongaro et al. (2008a) at Vesuvius. We therefore recommend avoidance of application of the energy-line method to stratified PDCs produced by partial column collapse, especially for hazard assessment purposes.

### Drawing PDC hazard maps from simulation outputs

**Fig. 11:**
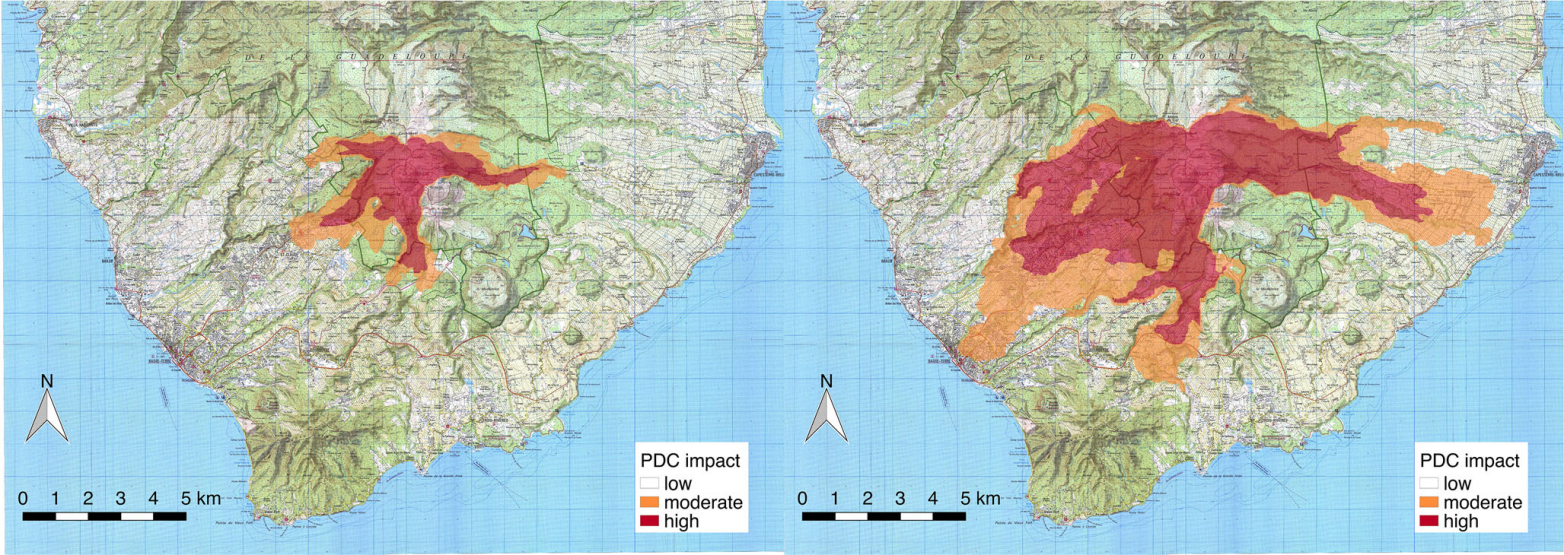
Maps of PDC hazard based on temperature isolines (300 K or 27 °C, orange; 600 K or 327 °C, red) for two scenarios of total column collapse with a mass eruption rate of 7 × 10^6^ kg/s (SP3, **a**) and 3 × 10^7^ kg/s (SP4, **b**)

Drawing hazard maps for a single scenario, based on numerical model results still is a challenging task that cannot be performed in a fully automatic way. It needs, instead, some *expert judgement* to account for model uncertainties (Calder et al. 2015). In particular, we have to consider the uncertainty associated with numerical errors and incomplete physical description of the phenomenon. Here, we have used different isolines of temperature to identify the areas reached by the most concentrated, basal part of the current and by the dilute ash cloud. In Fig. 11, we propose a preliminary identification of two hazard regions based on temperature isolines for both simulations SP3 and SP4. The 600 K isoline (327 °C) is considered as the region *very likely invaded* and *highly impacted by PDCs*, for the selected scenario. This is based on the following considerations:

- The isoline is stable and stationary in time (it does not further advance, once it reaches the displayed limit);
- This temperature threshold almost coincides with the limit of significant dynamic pressures (i.e. > 1 kPa) and of the maximum distance reached by the more concentrated (particle volume concentration of > 10^−3^) basal layer;
- Its position corresponds quite well with the limit of satisfactory vertical discretization (> 5 cells in the boundary layer) of the stratified PDC.

The 300 K (27 °C) isoline, on the other hand, encloses an area *susceptible to PDC invasion* and *moderately impacted*. In this area, simulated PDCs are mostly dilute, have temperatures of between 27 and 327 °C (still capable to cause severe injuries; Baxter et al. 2005, 2017), and have dynamic pressures of lower than 1 kPa. Here, however, the numerical uncertainty on the prediction is much larger and is more influenced by the physical approximations of the model (mostly, incomplete description) and by approximate boundary conditions at the ground surface.

Finally, the area outside of the 300 K (27 °C) isoline should be considered as *unlikely to be invaded* or *marginally impacted by PDCs* in the selected scenario, mostly because it is sheltered by significant topographic barriers or because of the great distance between the source and the location. However, it is important to note that different vent location, geometry, and eruption conditions, as well as atmospheric conditions, could potentially change the results for such a deterministic scenario. Therefore, the zonation presented here should be considered as a preliminary product to be refined in the future.

**Fig. 12:**
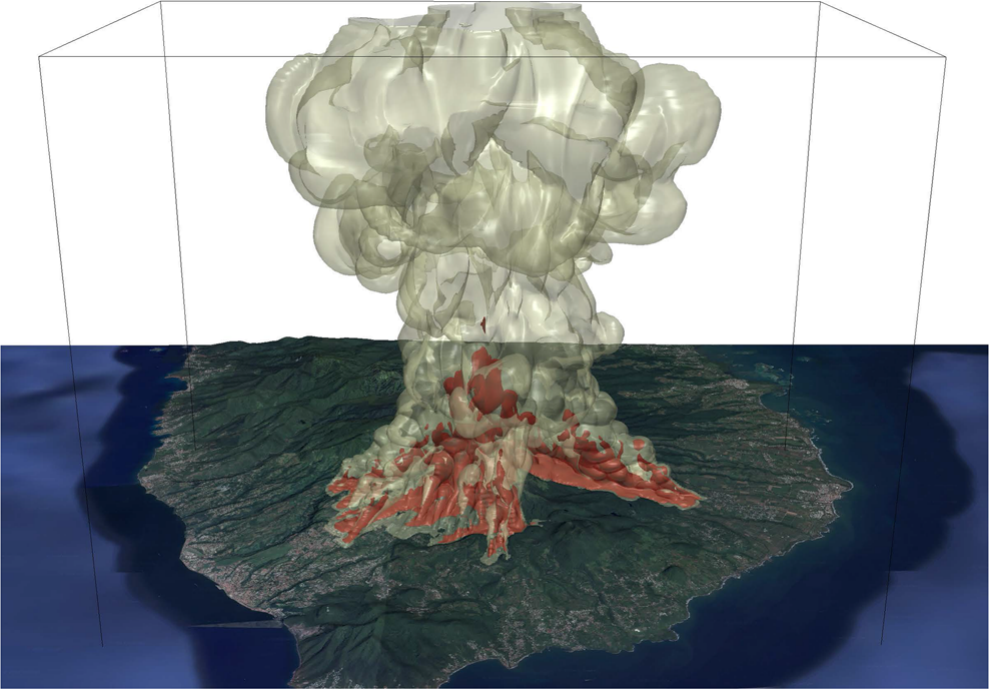
Example of visualization of 3D results for communication purposes. Simulation SP4 of total (> 90%) collapse, with increased mass eruption rate of about 2.8 × 10^7^ kg/s (run SP4), 400 s after the beginning of the collapse phase. Isosurfaces of 10^−7^ (outer, light green) and 10^−5^ (inner, brown) for the volume concentration of the fine ash are superimposed on a DEM of the volcano draped with satellite images (Google ©2018 Maxar Technology)

The use of clear and quantitative hazard maps for an individual scenario, in combination with three-dimensional visualization techniques (Fig. 12), can provide the tools for a better evaluation and communication of the hazards associated with a future scenario of a subplinian eruption at SDG, and contribute to a more effective risk management strategy. To aid with this, our Electronic Supplementary Material presents video animations for the development of a subplinian column and PDCs in scenario SP4, looking from the South-West (PDC branch moving towards the town of *St Claude;* Online Resource 1) and looking from the South (PDC branch moving towards the town of *Capesterre;* Online Resource 2).

The use of three-dimensional, multiphase flow models for PDC hazard assessment remains in any case challenging. The PDC hazard maps presented in Fig. 11 are limited to a single scenario (i.e. they are *conditional* maps), and because of the large computational cost of simulations (each lasting days to weeks on computer clusters with hundreds of CPU cores), it is difficult to assess model-related uncertainty. In addition, two key limitations in the physical/numerical description currently hinder a comprehensive simulation of PDCs. These are, first, the difficulty of describing the granular flow rheology across a broad range of particle concentrations, and second, the difficulty of achieving a vertical resolution fine enough to resolve the flow profile near the ground. The alternative use of simplified PDC models is also problematic, because the uncertainty associated to oversimplified boundary conditions (i.e. inability to describe the complexity of the explosive source), poorly constrained empirical parameters, and model approximations is even larger, although the low computational cost makes it possible to perform thousands of simulations to explore the parameter ranges in a probabilistic framework (Dalbey et al. 2008; Procter et al. 2010; Neri et al. 2015a, b; Ogburn and Calder 2017; Sandri et al. 2018; Rutarindwa et al. 2019; Lube et al. 2020). However, the exploitation of massive supercomputers and high-performance computing techniques, and the availability of open-source and community software, are driving a new step forward towards quantitative, probabilistic hazard assessment using 3D multiphase flow models. To this end, rigorous benchmarking studies are in progress to quantify the uncertainty associated with different model approximations and to provide a consensual metric for model validation (Esposti Ongaro et al. 2020).

## Conclusions

We have presented the results of a numerical study aimed at assessing the factors controlling propagation, emplacement, and hazards of PDCs in a credible (cf. Baxter et al. 2008) subplinian eruption scenario at La Soufrière de Guadeloupe. A set of deterministic simulations were constrained using the best estimates of eruption source parameters. One of the outcomes is that, even with a narrow range of mass eruption rates, subplinian eruptions can display very different eruptive styles, with different impacts from associated PDCs. Although exploration of a more extended range of eruptive conditions and a systematic appraisal of uncertainties would be necessary to perform a complete hazard assessment study, present results allow us to draw some preliminary conclusions and to contribute to the assessment of hazard associated with a potential future reawakening of the volcano.

Low-intensity (7 × 10^6^ kg/s) subplinian plumes are able to generate an oscillating column and steeply stratified PDC by a mechanism of partial collapse (50 to 70% of mass collapsing). Despite their ability to surmount proximal topographic barriers, PDC runout would be limited to the first 2–3 km from the vent and impacts on the inhabited region would be negligible. It is however possible (but not addressed by our modelling) that such weak plumes could be influenced by strong winds or asymmetric vent conditions, enhancing PDC runout in certain sectors.

Although partial collapse episodes are on average isotropic, the distribution of PDCs is asymmetric, due to the strong topographic control at the horseshoe-shaped collapse structure in the summit area. All simulations show that, given the present morphology of the La Soufrière de Guadeloupe volcano, most of the PDC mass will be focussed to the East-Northeast (which will take between 25 and 30% of the total mass), West-Southwest (between 25 and 50%), and South (10 and 15%), with a smaller portion (less than 5%) being emplaced in the North-Northwest sector. Between 5 and 30% will remain within the limits of the summit area.

Fully collapsing (fountaining or *boiling over*) conditions (90% of collapse) can be generated by a sudden enlargement of the vent (e.g. by a syn-eruptive partial edifice collapse such as that which occurred in the 1530 CE eruption), with a consequent reduction of the average exit velocity (at the same mass eruption rate) and air entrainment. In such a case, PDC intensity (mass flow rate per unit of angle), mobility (including capacity of surmounting topographic barriers), and the consequent impact on surrounding populations can be strongly enhanced, potentially affecting the inhabited regions > 4 km from the vent.

Increase in the mass flow rate at the vent to about 2.8 × 10^7^ kg/s (or funnelling of the collapsing mass into a single sector) is sufficient to generate more mobile PDCs that are able to reach the inhabited regions about 6 km from the vent. This is particularly the case as PDC mass is focussed in deep valleys and canyons (> 100 m deep) that reach far into the inhabited areas. Pyroclastic density currents with dynamic pressures exceeding 3 kPa and temperatures exceeding 200 °C can be expected in such cases and this is sufficient to inflict considerable damage to buildings and will be lethal to humans and animals (Baxter et al. 2005). Following Jenkins et al. (2013a), we will thus use, in a future study, the resulting spatial distribution of peak temperatures and dynamic pressures to develop a quantitative impact model for the population, infrastructure, and communication/facility networks. This will be combined with vulnerability information derived from medical analyses (cf. Baxter et al. 2017) and building engineering (cf. Jenkins et al. 2013b), and with exposure data, to quantify the risk.

Finally, by combining information on the spatial distribution of temperature and dynamic pressure with objective considerations regarding model-related uncertainty, we are able to draw preliminary PDC hazard maps for a subplinian eruptive scenario. This still requires some level of expert judgement to identify the factors that control uncertainty of numerical simulation results. In such a representation, and for the reference subplinian scenario, we identify three areas varying in susceptibility to invasion by PDC: *very likely to be invaded* (with dynamic pressures of > 1–10 kPa and temperatures of > 300 °C), *susceptible to invasion* (with lower dynamic pressures and temperatures), and *unlikely to be invaded* by PDCs. This information will need to be updated in the future by considering a broader set of eruptive conditions and uncertainties. However, we believe that, given the current increasing unrest, it can provide a useful and timely contribution to hazard assessment and crisis response in the advent of a future eruption at La Soufrière de Guadeloupe, while being a blueprint as to how to set-up hazard maps for subplinian eruption scenarios elsewhere.

### Supplementary Information

ESM 1 (AVI 1037 kb)

ESM 2 (AVI 1011 kb)

## Figures and Tables

**Fig. 13:**
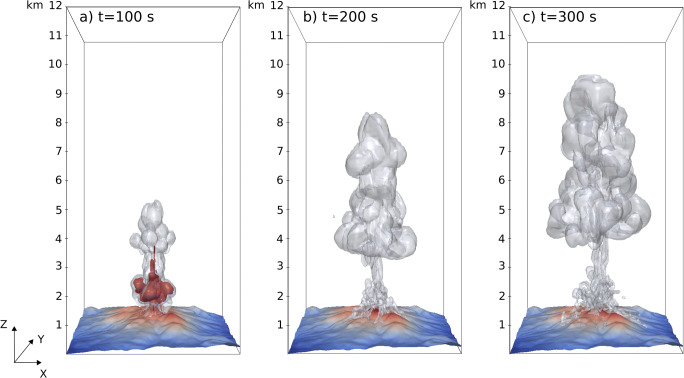
Isosurface at 10^−5^ and 10^−7^ of total particle concentration in the atmosphere, at **a**) 100 s, **b**) 200 s and **c**) 300 s after the beginning of the collapse phase. The flow at the conduit exit has been stopped after 20 s, when the jet has reached its maximum thrust height of about 2 km above the vent. The *X*-axis is oriented West-East, *Y* is oriented South-North

**Fig. 14:**
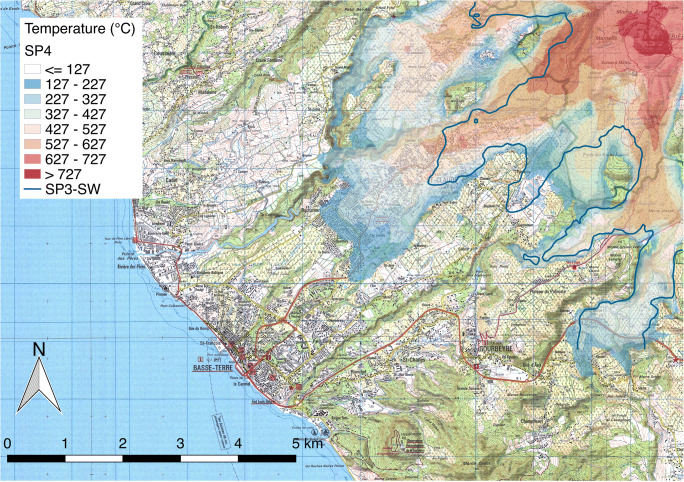
Comparison between run SP4 and run SP3-SW at *t *= 700 s. Mixture temperature in the first computational cell above the topography (representing the averaged value over the first 20 m). The color map resulting from SP4 numerical simulation has been superimposed on the IGN map. The blue line indicates the 127 °C temperature isoline for simulation SP3-SW

## Appendix

### Appendix 1. Physical and numerical model

The PDAC (Pyroclastic Dispersal Analysis Code) model solves the coupled transport equations of mass, momentum, and enthalpy for a mixture of volcanic gas, atmospheric air, and three particle classes representative of fine to coarse ash. The Eulerian-Eulerian multiphase flow model formulation from Neri et al. (2003) has been adopted for gas and three single particulate classes, representing fine to coarse ash. For the four-phase gas-particle mixture, the following 3D transport equations for mass, momentum, and energy are solved. Gas and particle equations are coupled by the drag forces (allowing to simulate settling, sedimentation, and preferential concentration) and thermal exchange.

**Mass equations**

$$ {\displaystyle \begin{array}{c}\frac{\partial \left({\alpha}_g{\rho}_g\right)}{\partial t}+\nabla \cdot \left({\alpha}_g{\rho}_g{\boldsymbol{U}}_g\right)=0\\ {}\frac{\partial \left({\alpha}_s{\rho}_s\right)}{\partial t}+\nabla \cdot \left({\alpha}_s{\rho}_s{\boldsymbol{U}}_s\right)=0;s=1,\dots, N\end{array}} $$

**Momentum equations**

Because particle-particle collisions are neglected in the solid stress tensor, the solid pressure term is not included in the particle momentum equation (Model A of Gidaspow 1994).

$$ {\displaystyle \begin{array}{c}\frac{\partial }{\partial t}\left[{\alpha}_g{\rho}_g{\boldsymbol{U}}_g\right]+\nabla \cdot \left[{\alpha}_g{\rho}_g{\boldsymbol{U}}_g{\boldsymbol{U}}_g\right]=-{\alpha}_g\nabla P-\nabla \cdot {\boldsymbol{T}}_g+{\alpha}_g{\rho}_g\boldsymbol{g}+{\sum}_{s=1}^N{D}_{gs}\left({\boldsymbol{U}}_s-{\boldsymbol{U}}_g\right)\\ {}\frac{\partial }{\partial t}\left[{\alpha}_s{\rho}_s{\boldsymbol{U}}_s\right]+\nabla \cdot \left[{\alpha}_s{\rho}_s{\boldsymbol{U}}_s{\boldsymbol{U}}_s\right]=-{\alpha}_s\nabla P-\nabla \cdot {\boldsymbol{T}}_s+{\alpha}_s{\rho}_s\boldsymbol{g}-{D}_{gs}\left({\boldsymbol{U}}_s-{\boldsymbol{U}}_g\right)+{\sum}_{p=1p\ne s}^N{D}_{ps}\left({\boldsymbol{U}}_s-{\boldsymbol{U}}_p\right);s=1,\dots, N\end{array}} $$

**Enthalpy equations**

The total enthalpy of the *i*th phase is defined as the sum ${h}_i={e}_i+\frac{P_i}{\rho_i}$, with *e*_*i*_ as the specific internal energy. The work done by gravity and by the drag and viscous forces is neglected in both balances. Heat exchange between solid particles is also neglected, following Neri et al. (2003) and previous studies on analogous eruption scenarios.

$$ {\displaystyle \begin{array}{c}\frac{\partial }{\partial t}\left[{\alpha}_g{\rho}_g{h}_g\right]+\nabla \cdot \left[{\alpha}_g{\rho}_g{h}_g{\boldsymbol{U}}_g\right]=-\nabla \cdot \left({\alpha}_g{\boldsymbol{q}}_g\right)-{\alpha}_g\left[\frac{\partial {P}_g}{\partial t}+{\boldsymbol{U}}_g\cdot \nabla {P}_g\right]+{K}_{gs}\left({T}_s-{T}_g\right)\\ {}\frac{\partial }{\partial t}\left[{\alpha}_s{\rho}_s{h}_s\right]+\nabla \cdot \left[{\alpha}_s{\rho}_s{h}_s{\boldsymbol{U}}_s\right]=-\nabla \cdot \left({\alpha}_s{\boldsymbol{q}}_s\right)+{K}_{gs}\left({T}_g-{T}_s\right);s=1,\dots, N\end{array}} $$

**Closure equations**

The gas and solid phases are treated as interpenetrating continua. Their volume fractions obey the closure equation:

$$ {\alpha}_g+\sum \limits_{s=1}^N{\alpha}_s=1 $$

The thermal equation of state of the gas phase is that of perfect gases. Particle density is constant.

$$ {\rho}_g={\rho}_g\left(P,{T}_g\right);{\rho}_s=\mathrm{constant} $$

For both the gas and particles, the stress tensor is written in a Newtonian form:

$$ {\displaystyle \begin{array}{c}{\boldsymbol{T}}_i={\alpha}_i{\rho}_i\left[2{\nu}_{\mathrm{eff}}{\boldsymbol{S}}_i+{\lambda}_i\left(\nabla \cdot {\boldsymbol{U}}_i\right)\boldsymbol{I}\right]\\ {}{\boldsymbol{S}}_i=\frac{1}{2}\left(\nabla {\boldsymbol{U}}_i+\nabla {{\boldsymbol{U}}_i}^T\right)-\frac{1}{3}\left(\nabla \cdot {\boldsymbol{U}}_i\right)\boldsymbol{I}\end{array}} $$

where the subscript (*i*) indicates either gas or particles and *ν*_eff_ is the effective viscosity coefficient (*ν*_eff_ = *ν* + *ν*_turb_) and is the sum of the molecular/granular viscosity *ν* and the turbulent (sub-grid) turbulent coefficient. For the gas, the molecular viscosity depends on temperature and is equal to 1.84 × 10^−5^ for gas at ambient temperature; the turbulent term is defined by Smagorinsky’s Large-Eddy Simulation model (Neri et al. 2003). For particles, the granular viscosity is proportional to particle concentration *ν*_*s*_ = *c*_*s*_*α*_*s*_ (Miller and Gidaspow 1992), whereas the turbulent-collisional turbulent stress is neglected.

The heat flux is expressed through the Fourier law, for both gas and particles:

$$ {\boldsymbol{q}}_i=-{\rho}_i\left({D}_i+\frac{\nu_{\mathrm{turb}}}{\mathit{\Pr}}\right){C}_{P,i}\nabla {T}_i $$

where Pr is the turbulent Prandtl number, set equal to 0.7 for gas and 0 for particles.

Finally, the gas-particle drag coefficient *D*_*gs*_ is expressed by the Ergun-Wen-Yu law, whereas the gas-particle heat transfer coefficient *K*_*ht*_ is expressed by the Ranz-Marshall law. We refer to Neri et al. (2003) for model details.

Initial conditions represent a standard, stratified dry atmosphere. Free inflow/outflow conditions are imposed at the top, and lateral boundaries, no-slip, and zero gradient conditions are imposed at the ground. Inlet conditions are imposed on a circular vent, where all fluid fields are imposed: the volume fraction, velocity, and temperature of each particle class, and the pressure, velocity, and temperature of the gas, plus the mass fraction of water vapour. The fields are corrected to account for the correct mass flow rate when the circular vent is discretized on a Cartesian mesh.

Details on the numerical solution algorithm are given by Esposti Ongaro et al. (2007). Table 3 reports the complete list of performed numerical sensitivity tests.

**Table 3:** List of performed numerical simulations, with different numerical mesh resolutions. Symbol (§) indicates the simulations described in the paper. Symbol (*) indicates that the same simulations have been repeated with/without immersed boundary conditions (de’ Michieli Vitturi et al. 2007). Symbol (†) indicates that the same simulation has been repeated with modified ground boundary condition (a leaky boundary, as in Dufek and Bergantz 2007b)

Numerical parameters	*Domain* [km^3^]	*Number of cells* (nx × ny × nz)	*Minimum-Maximum grid size dx-dy* [m]	*Minimum-Maximum grid size dz* [m]
*SP1*	6 × 6 × 12 (§,†,*)	200 × 200 × 200	10–50	10–200
	6 × 6 × 8	200 × 200 × 350	10–50	5–100
	11 × 11 × 8	200 × 200 × 250	10–100	10–100
*SP2*	6 × 6 × 12 (†)	200 × 200 × 200	10–50	10–200
	11 × 11 × 8	100 × 100 × 150	20–250	10–200
	11 × 11 × 8 (§)	200 × 200 × 150	10–50	10–200
*SP3*	6 × 6 × 12	200 × 200 × 200	10–50	10–200
	20 × 12 × 8 (§)	400 × 200 × 150	20–100	20–200
*SP3-SW*	6 × 6 × 8	200 × 200 × 200	10–50	10–100
		100 × 100 × 150	20–100	20–100
*SP3-SE*	8 × 11 × 8	120 × 150 × 150	20–100	20–100
*SP4*	20 × 12 × 15 (§)	400 × 200 × 200	20–100	20–200

**Table 4:** List of mathematical symbols (bold indicates vectors or tensors)

Symbol	Definition	Units
***α***	Phase volume fraction	
***ρ***	Density	kg m^−3^
***U***	Velocity (vector)	m s^−1^
P	Pressure	kg m^−1^ s^−2^
T	Temperature	K
h	Specific enthalpy	m^2^ s^−2^
D_*gs*_	Gas-particle drag coefficient	kg m^−3^ s^−1^
D_*ps*_	Particle-particle drag coefficient	kg m^−3^ s^−1^
K_*gs*_	Gas-particle heat exchange coefficient	m^2^ s^−3^ K^−1^
***T***	Deviatoric stress tensor	kg m^−1^ s^−2^
***S***	Deviatoric strain	s^−1^
***I***	Identity tensor	
***q***	Heat flux	kg s^−3^
D_*i*_	Diffusion coefficient	
*ν,ν*_*turb*_*,ν*_*eff*_	Molecular, turbulent, and effective kinematic viscosity	m^2^ s^−1^
*λ*	Second molecular viscosity	m^2^ s^−1^
Pr	Prandtl number	
*C*_*P,i*_	Specific heat at constant Pressure	m^2^ s^−2^ K^−1^
***g***	Gravity acceleration	m s^−2^
*g,s,p*	Subscripts for gas and solid phases	
*N*	Total number of gas + solid phases	

## Appendix 2. Single collapse pulse

To better understand the mechanism of PDC formation by partial column collapse (50% of mass collapsing), we have simulated a single, impulsive collapse scenario, by stopping the flow feeding at the volcanic vent at the time where the column had reached its maximum thrust height (at *t* = 20 s). In this way, we avoided the complexity associated to the superposition of multiple collapse events in the caldera and, consequently, reduce the problem of the accumulation of pyroclasts in the basal flow to form a dense basal layer.

At 20 s, the total erupted mass was equal to about 1.4 × 10^8^ kg. The distribution of the total particle volumetric fraction in the atmosphere is represented in Fig. 13 by the two isosurfaces at 10^−4^ and 10^−6^ (Fig. 4 presents the same figure but for a continuous feeding). The invaded area is reduced, with respect to the case where the column is continuously fed. This observation confirms that accumulation of pyroclasts in the proximal area is a key element to understand PDC mobility and to correctly predict their interaction with the volcano morphology (including channelization effects) and their final runout.

## Appendix 3. Anisotropic PDC focussing in the South-West sector

Simulations were performed towards the most inhabited region, to the South-Western sector of the volcano, where most of the collapsing mass is driven by the proximal caldera morphology (Fig. 14). We imposed the same mass eruption rate of scenario SP3 on one quarter of the domain (i.e. focussing in a 90° sector), which is equivalent to a mass eruption rate 4 times larger (i.e. 2.8 × 10^7^ kg/s), with a consequently larger impact. Because the mass flow rate per unit of angle is the same as the SP4 case, the resulting PDC runout and intensity in the inhabited region of St. Claude are comparable. However, simulation SP4 has a much longer runout to the South-West, as a result of anisotropic mass distribution. As visible in Fig. 9b, the percentage of mass conveyed towards South-West in run SP4 is larger than 35% of the total collapsing mass, which, alone, is larger than the total mass flow rate in run SP3-SW.
